# Quantitative High-Throughput Profiling of Environmental Chemicals and Drugs that Modulate Farnesoid X Receptor

**DOI:** 10.1038/srep06437

**Published:** 2014-09-26

**Authors:** Chia-Wen Hsu, Jinghua Zhao, Ruili Huang, Jui-Hua Hsieh, Jon Hamm, Xiaoqing Chang, Keith Houck, Menghang Xia

**Affiliations:** 1 National Center for Advancing Translational Sciences, National Institutes of Health, Bethesda, MD; 2 Division of the National Toxicology Program, National Institute of Environmental Health Sciences, National Institutes of Health, Research Triangle Park, NC; 3 Integrated Laboratory Systems, Inc., Morrisville, NC; 4 U.S. Environmental Protection Agency, Research Triangle Park, NC

## Abstract

The farnesoid X receptor (FXR) regulates the homeostasis of bile acids, lipids, and glucose. Because endogenous chemicals bind and activate FXR, it is important to examine which xenobiotic compounds would disrupt normal receptor function. We used a cell-based human FXR β-lactamase (*Bla*) reporter gene assay to profile the Tox21 10K compound collection of environmental chemicals and drugs. Structure-activity relationships of FXR-active compounds revealed by this screening were then compared against the androgen receptor, estrogen receptor α, peroxisome proliferator-activated receptors δ and γ, and the vitamin D receptor. We identified several FXR-active structural classes including anthracyclines, benzimidazoles, dihydropyridines, pyrethroids, retinoic acids, and vinca alkaloids. Microtubule inhibitors potently decreased FXR reporter gene activity. Pyrethroids specifically antagonized FXR transactivation. Anthracyclines affected reporter activity in all tested assays, suggesting non-specific activity. These results provide important information to prioritize chemicals for further investigation, and suggest possible modes of action of compounds in FXR signaling.

The farnesoid X receptor (FXR), a bile acid-activated nuclear receptor, plays a crucial role in maintaining the homeostasis of bile acids, lipids, and glucose^1^,^2^. FXR contains a DNA-binding domain for docking to target genes with an FXR response element (FXRE) and a ligand-binding domain (LBD) used by intracellular ligands. Binding of FXR agonists to FXR-LBD induces conformational changes in FXR and promotes expression of target genes including small heterodimer partner (SHP) and bile salt export pump (BSEP)^3^. Activation of SHP results in repression of two key cytochrome P450 enzymes in bile acid biosynthesis, cholesterol 7-alpha-monoxygenase (CYP7A1) and 12-alpha-hydroxylase (CYP8B1), as well as several important regulators of glucose metabolism including glucose 6-phosphatase (G6Pase), fructose-1,6-bisphosphatase 1 (FBP1), and phosphoenolpyruvate carboxykinase (PEPCK)^4^. The well-characterized endogenous ligands of FXR are bile acids including chenodeoxycholic acid (CDCA), cholic acid (CA), deoxycholic acid (DCA), lithocholic acid (LCA), and ursodeoxycholic acid (UDCA)^5^,^6^. Guggulsterone, a plant steroid, was the first natural FXR antagonist identified^7^,^8^,^9^. Other FXR ligands include natural products and investigational drugs. Ivermectin, an avermectin antiparasitic, has been recently identified to bind FXR-LBD and decrease serum glucose and cholesterol levels in mice^10^. Synthetic FXR agonists including GW4064, INT-747, PX-102, FXR-450, and their analogs are under development for treating dyslipidemia, diabetes, primary biliary cirrhosis (PBC), and nonalcoholic steatohepatitis (NASH)^11^,^12^. GW4064, an isoxazole derivative that selectively activates FXR at submicromolar potencies, has been shown to lower serum triglyceride levels in rats^13^ but to have limited clinical uses caused by poor bioavailability, fast metabolism, and toxicity at high doses^14^. INT-747, so called obeticholic acid (OCA) or 6-alpha-ethyl chenodeoxycholic acid (6-ECDCA), is a 6-alpha-alkyl-substituted analog of CDCA selectively inducing FXR transactivation at 1 μM^15^. INT-747 is being tested as a monotherapy in a Phase 3 clinical trial and as a combination therapy with UDCA in a Phase 2 clinical trial for treating patients with PBC (ClinicalTrial.gov identifier: NCT01473524 and NCT00550862). PX-102 or PX20606^16^, a non-steroidal FXR agonist developed to treat NASH, showed safety and good tolerance in a Phase 1 trial (ClinicalTrial.Gov identifier: NCT1998659 and NCT1998672). FXR-450, or so called WAY-362450, is an azepino[4,5-b]indole-based FXR agonist capable of lowing plasma triglycerides and toal cholesterol levels in a dyslipidemia model^17^.

Despite the growing interest in FXR ligands in drug discovery, little is known with regard to the roles of FXR in mediating or protecting xenobiotic-induced toxicity. Abnormal FXR function leads to numerous disorders such as cholestasis, diabetes, and cancer, and plays a role in liver regeneration^18^. Depending on the dose of FXR ligands, distinct outcomes have been observed across different species. Exposure of GW4064 has been reported to cause hepatobiliary injury to medaka eleutheroembryos^19^. CA and GW4064 have been found to protect mice from acetaminophen-induced hepatotoxicity^20^,^21^ yet a CDCA-rich diet has been reported to induce liver hypertrophy in mice^22^. Treatment with the FXR antagonist tempol or intestine-specific deletion of FXR led to similar anti-obesity effects in mice^23^. Theonellasterol, a recently discovered FXR antagonist from marine sponge, protected mice susceptible to cholestasis from bile acid-induced liver damage^24^. In addition, gastric bypass surgery has emerged as a potential therapy for diabetes mellitus type 2^25^ where FXR-dependent increase of circulating total bile acids was observed in mice treated with vertical sleeve gastrectomy^26^. These studies suggest that agonists, antagonists, and modulators of FXR could exert protective or adverse effects depending on health states and exposure doses.

One challenge in defining the role of FXR in mediating xenobiotic-induced toxicity is the depth of data on the structural classes of chemicals that act on FXR. Here, we report the profiling of 10,766 substances (8599 unique compounds) in modifying FXR signaling and associated cytotoxicity as part of the Tox21 Phase II program^27^. We utilized the quantitative high-throughput screening (qHTS) data combined with computational methods to identify biological activity patterns of the Tox21 10K compound collection in order to prioritize chemicals for more extensive follow-up studies. Compounds identified as FXR-actives were grouped into several clusters based on similarities in chemical structure, drug class, or known biological target. The representative FXR-active clusters were further compared for their selectivity against other tested human nuclear receptors including the androgen receptor (AR), estrogen receptor alpha (ERα), peroxisome proliferator-activated receptor delta (PPARδ), peroxisome proliferator-activated receptor gamma (PPARγ), and the vitamin D receptor (VDR) to identify FXR-specific chemical scaffolds.

## Results

### qHTS performance of FXR-*bla* and viability assays

**Figure 1:**
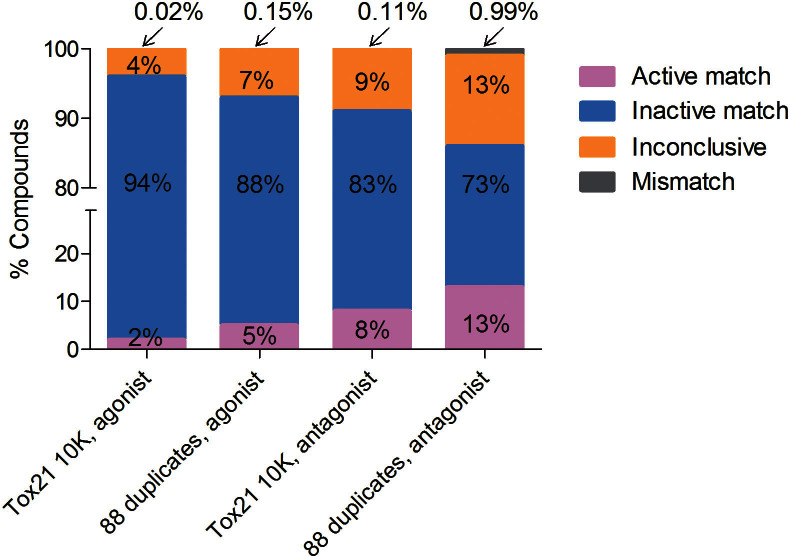
Reproducibility of FXR qHTS data. Data reproducibility of the triplicate run of the Tox21 10K compounds and the 88 replicated compounds in the primary screening of the FXR-*bla* assay. Data reproducibility is measured by the fraction of active match, inactive, mismatch and inconclusive cases.

To identify environmental chemicals and drugs that modulate FXR signaling, we screened the Tox21 10K compound library against the FXR-*bla* assay in both agonist and antagonist modes. To rule out FXR antagonist response caused by compound cytotoxicity, a cell viability assay was conducted in the same well as the FXR-*bla* assay. The ratiometric readouts of FXR-*bla* assay for measuring FXR activity are based on the β-lactamase-coupled fluorescence resonance energy transfer (FRET) technology^28^. CDCA and (Z)-guggulsterone, positive controls for agonist and antagonist screening, respectively, yielded an EC_50_ value [i.e. concentration calculated to induce a half maximal response with standard deviation (SD)] of 29 ± 6 μM and an IC_50_ value (i.e. concentration calculated to inhibit a half maximal response with SD) of 50 ± 12 μM. The agonist and antagonist screening worked well as evaluated by average signal-to-background (S/B) ratios of 4.4 for both assays, and average coefficients of variation (CV) of 7.0% and 3.5%, respectively. The average Z′ factors of the agonist and antagonist screening were 0.35 and 0.75, respectively. Cytotoxicity screening in the FXR agonist and antagonist screening also showed consistent responses with average S/B ratios of 67.1 and 67.7, average CV values of 13.0% and 12.0%, and average Z′ factors of 0.60 and 0.69, respectively. Data reproducibility of a given compound was assigned as active agonist/antagonist match, inactive match, inconclusive, or mismatch based on average curve rank and percentage of inactive outcomes of the three independent measurements^28^. The triplicate runs of the Tox21 10K compound collection as well as the 88 compounds duplicated in each plate showed low mismatch rates of <1% in the FXR-*bla* screening (Figure 1). The antagonist and agonist screening identified 8% (861 substances) and 2% (215 substances) active matches, respectively, containing FXR-active compounds and positives resulting from assay artifacts.

### Identification of FXR agonists and antagonists

**Figure 2:**
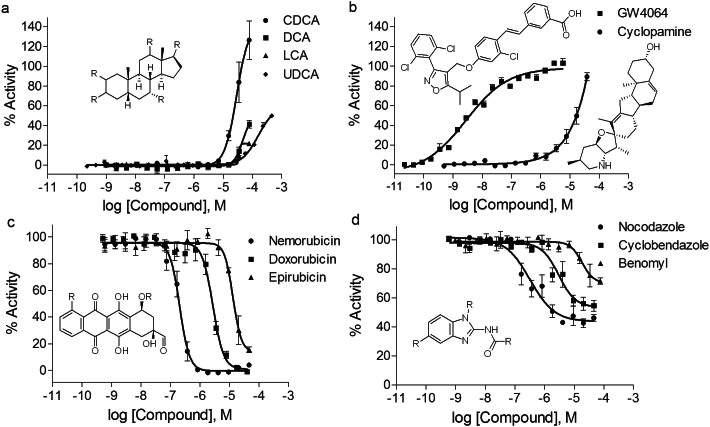
Concentration response curves of selective compounds identified by the FXR-*bla* assay. Concentration response curves of (a) cholic acids: CDCA (EC_50_ = 28.62 μM), DCA (EC_50_ = 47.31 μM), LCA (EC_50_ = 34.90 μM), and UDCA (EC_50_ = 120.70 μM) in agonist mode; (b) GW4064 (EC_50_ = 0.003 μM) and cyclopamine (EC_50_ = 10.57 μM) in agonist mode; (c) anthracyclines: nemorubicin (IC_50_ = 0.21 μM), doxorubicin (IC_50_ = 2.74 μM), and epirubicin (IC_50_ = 13.10 μM) in antagonist mode; (d) benzimidazoles: nocodazole (IC_50_ = 0.32 μM), cyclobendazole (IC_50_ = 3.00 μM), and benomyl (IC_50_ = 18.28 μM) in antagonist mode. Data were collected from primary screening and expressed as mean ± SD from 3 experiments.

After the primary screening, the test compounds were categorized as active agonists/antagonists, inconclusive, or inactive compounds based on the activities observed in both ratiometric and 460 nm readings^28^. There were 1141 and 2172 compounds that showed activities in the FXR-*bla* agonist and antagonist mode assays, respectively. Four known FXR agonists, CDCA (EC_50_ = 28.62 μM), DCA (EC_50_ = 47.31 μM), GW4064 (EC_50_ = 0.003 μM), and UDCA (EC_50_ = 120.70 μM) as well as two well-characterized FXR antagonists, (*E*)-guggulsterone (IC_50_ = 24.06 μM) and (*Z*)-guggulsterone (IC_50_ = 39.05 μM), were identified from the screening (Table S1, Figure 2a, Figure 2b). Some positive compounds were further verified and re-assigned as inconclusive compounds by additional criteria to exclude potential false positives as a result of compound autofluorescence (efficacy_FXR-bla 460 nm_/efficacy_FXR-bla ratio_ or efficacy_FXR-bla, 535 nm_/efficacy_FXR-bla, ratio_ > 2, PubChem assay identifier: 720681 and 720682) or cytotoxicity (IC_50, viability_/IC_50, FXR-bla ratio_ < 3, p < 0.05). For example, benzo(*k*)fluoranthene and triamterene are highly fluorescent at 460 nm in the assay medium, producing a concentration-dependent increase in all β-lactamase-based assays (data not shown). Digoxin and bortezomib were inconclusive antagonists of FXR because the two compounds showed FXR antagonist activity at or near cytotoxic concentrations (Table S1). Two hundreds and sixty-six unique compounds including potent FXR-active compounds (EC_50_ or IC_50_ values < 10 μM) identified from the primary screening and selected structural analogs were re-tested in the same FXR-*bla* and viability assays, yielding confirmation rates of 67% (73 of 109) and 90% (144 of 160) in the agonist and antagonist screening, respectively. Twenty-five novel and representative compounds with agonist or antagonist activities confirmed in the FXR-*bla* assay were shown in (Table 1) detailing compound efficacy, potency, curve class, and data reproducibility in the primary and confirmatory screening. The 25 compounds were further tested in a FXR coactivator recruitment assay to determine whether a given FXR-active compounds is an FXR ligand or a potential FXR signaling modulators (Table 1). The agonist control CDCA showed an EC_50_ value of 29.90 μM in binding of FXR-LBD and inducing coactivator recruitment, and the known FXR ligand ivermectin fully inhibited CDCA-induced coactivator recruitment with an IC_50_ value of 0.91 μM. Cyclopamine (EC_50_ = 10.57 μM, efficacy = 94%) and 9-aminoacridine (EC_50_ = 11.17 μM, efficacy = 152%) showed full agonist activity, and both compounds were unable to induce coactivator recruitment to FXR-LBD (Table 1). Several partial FXR agonists including daunorubicin (EC_50_ = 1.02 μM, efficacy = 48%), doxorubicin (EC_50_ = 1.35 μM, efficacy = 68%) and epirubicin (EC_50_ = 5.78 μM, efficacy = 44%) also showed antagonist effects in the FXR-*bla* assay with IC_50_ values of 5.53 μM, 2.80 μM and 17.80 μM, respectively (Table 1). These FXR-active anthracyclines were able to inhibit CDCA-induced coactivator recruitment at potencies similar to their antagonist activity in the FXR-*bla* assay (Table 1). Among the confirmed compounds that completely inhibited CDCA-induced FXR-*bla* activity, actinomycin D (IC_50_ = 0.02 μM) was the most potent, followed by flavopiridol (IC_50_ = 0.02 μM), nemorubicin (IC_50_ = 0.13 μM), gimatecan (IC_50_ = 2.69 μM), and emetine (IC_50_ = 4.23 μM). Colchicine (IC_50_ = 0.03 μM, efficacy = 54%), nocodazole (IC_50_ = 0.29 μM, efficacy = 68%), picropodophyllin (IC_50_ = 0.02 μM, efficacy = 55%), and vinorelbine (IC_50_ = 0.03 μM, efficacy = 62%) caused partial inhibition of CDCA-induced FXR transactivation in both primary screening and confirmation studies (Table 1). Colchicine was also identified as an FXR antagonist in the coactivator recruitment assay with an IC_50_ value of 0.03 μM and 52% efficacy (Table 1).

### Structural clusters of FXR agonists and antagonists

**Figure 3:**
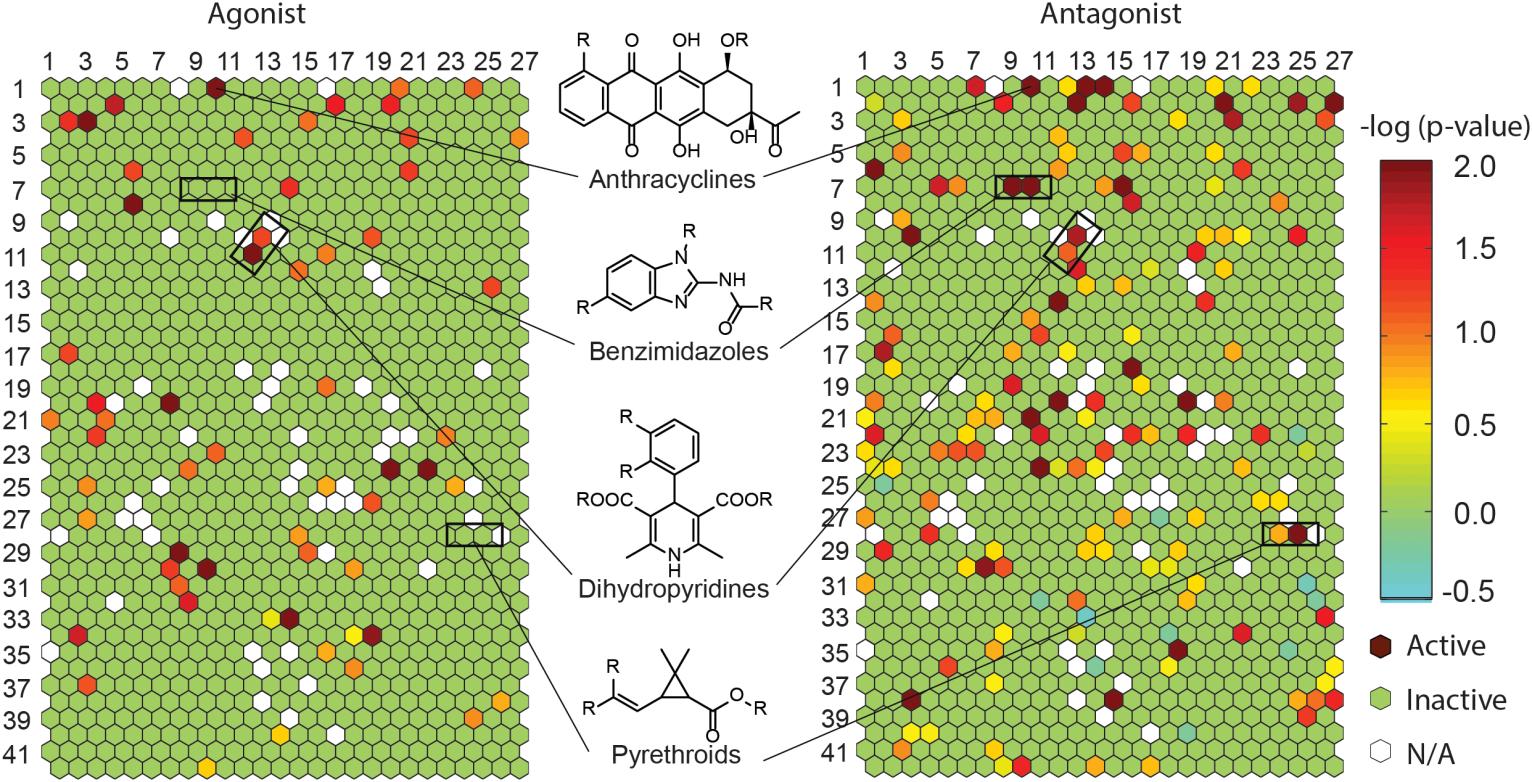
Heat maps of structural classes versus FXR activity. Each hexagon represents a cluster of structurally similar compounds. Clusters are annotated with a (x,y) coordinate and colored according to the enrichment of FXR actives [−log(p-value)] in the cluster. Warmer colors indicate higher enrichment of FXR actives and colder colors indicates less significant enrichment of FXR actives. Empty clusters are colored in white.

The Tox21 10K compound collection was first grouped into 1,014 clusters of structural classes using the self-organizing map (SOM) algorithm^29^. As shown in Figure 3, each cluster containing unique and replicated compounds was colored according to the significance of enrichment (negative logarithmic scale of a p-value) in FXR-active agonists or antagonists to assess the probability of a given chemical scaffold to activate FXR-*bla* or inhibit CDCA-induced FXR-*bla* transactivation. The FXR-active compounds yielded 189 structural classes in which 56 and 152 clusters were significantly enriched with compounds that activate or inhibit FXR, respectively. The representative FXR-active clusters are described in Table S2.

The clusters of FXR agonists include cholic acids (k39.24), avermectins (k3.3), and retinoic acids (k22.22 and k20.7). The cholic acid cluster (k39.24) contains three known FXR agonists, CDCA, DCA, and LCA. CDCA was two-fold and six-fold more efficacious than DCA and LCA in activating FXR (Figure 2a and Table S1), respectively. Avermectins (k3.3), which include abamectin, doramectin, milbemectin, and ivermectin, were identified as partial FXR agonists with EC_50_ values from 0.44 μM to 37.71 μM and maximum efficacy values of 27–90% (Table S1). Ivermectin also antagonized CDCA-induced FXR transactivation with an IC_50_ value of 2.50 μM. The retinoic acids (k22.22)-13-*cis* retinoic acid, tretinoin as well as the benzoic analogs AM80 and AM580 (k20.7) acted as FXR agonists and mixed agonists/antagonists, with EC_50_ values in the agonist mode that ranged from 24.61 μM to 51.11 μM (Table S1). However, moxidectin, a milbemycin derivative structurally similar to avermectins, did not show agonist activity in the FXR-*bla* assay (data not shown).

The anthracycline (k1.10) and the dihydropyridine (k10.12 and k11.12) clusters are enriched with both partial FXR agonists and FXR antagonists. All compounds in the anthracycline cluster except nemorubicin showed mixed FXR agonist/antagonist responses (Table 1 and Table S1). For example, nemorubicin, doxorubicin, and epirubicin inhibited CDCA-induced FXR transactivation at potencies ranging from 0.21 μM to 13.10 μM (Figure 2c). Ten of 19 unique dihydropyridine compounds (e.g., felodipine, lemildipine, nicardipine) were found to be partial FXR agonists (EC_50_ values of 3.64 μM to 28.90 μM). In contrast, benidipine (k10.12), nifedipine (k11.12), and nimodipine (k11.12) in the same structural classes showed FXR antagonist activities with IC_50_ values of 24.78 μM, 34.02 μM, and 31.06 μM, respectively.

Other clusters of potential FXR antagonists identified from the screening were vinca alkaloids (k2.26), benzimidazoles (k7.9 and k7.10), flavonoids (k1.13 and k2.12), estradiols (k20.11), and pyrethroids (k28.23 and k28.24) (Table S1). The vinca alkaloids (k2.26) including vincristine, vinblastine, and vinorelbine inhibited CDCA-induced FXR activation with IC_50_ values of 0.03 μM to 0.12 μM (Table 1). In the benzimidazole clusters (k7.9 and k7.10), 9 of 13 unique compounds including nocodazole, cyclobendazole, and benomyl acted as FXR antagonists (Figure 2d, Table 1 and Table S1). Several flavonoids including genistein (k1.13), biochanin A (k1.13), apigenin (k2.12), and chrysin (k2.12) showed partial to complete antagonistic response in the FXR-*bla* assay with IC_50_ values ranging from 12.07 μM to 46.19 μM (Table S1). Four of eight unique estradiol analogs (k20.11) including 17β-estradiol (IC_50_ = 33.15 μM) and 17α-ethinylestradiol (IC_50_ = 16.65 μM) exhibited antagonistic activity against FXR (Table S1). Twelve out of 17 unique pyrethroids (e.g., bifenthrin in k28.23, cyhalothrin in k28.24) were able to inhibit CDCA-induced FXR activation with IC_50_ values ranging from 9.36 μM to 33.90 μM (Table S1).

### Mechanism of action and targets in FXR signaling

Compounds which share similar biological functions despite distinct chemical scaffolds may be used to identify potential interactions between their biological target and FXR. Using the known primary targets and mechanism of action of approved and investigational drugs, 35 FXR-active drugs more potent than the natural FXR ligands [e.g., CDCA for agonists; (Z)-guggulsterone for antagonists] were classified into four drug classes and six target classes depending on their primary biological function (Table S3).

**Figure 4:**
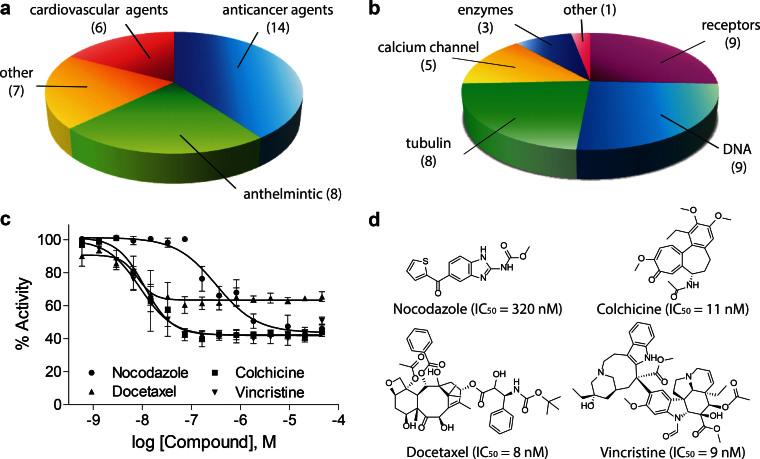
Distribution of biological functions of selective potent FXR-actives. Thirty-five FXR-active drugs with submicromolar potency and known biological targets were chosen to form clusters based on biological functions. (a) Distribution of drug classes. (b) Distribution of target classes. (c) Concentration-dependent inhibition curves of nocodazole (IC_50_ = 320 nM), colchicine (IC_50_ = 11 nM), docetaxel (IC_50_ = 8 nM), and vincristine (IC_50_ = 9 nM) measured from the primary screen. (d) Chemical structures of nocodazole, colchicine, docetaxel, and vincristine.

Four classes of FXR-active pharmaceuticals are anticancer, cardiovascular, anthelmintic, and miscellaneous drugs (Figure 4a). The largest drug class has 14 anticancer agents including five anthracyclines (daunorubicin, doxorubicin, epirubicin, idarubicin, and nemorubicin), three vinca alkaloids (vinblastine, vincristine, and vinorelbine), a benzimidazole (nocodazole), a synthetic flavonoid (flavopiridol), a lignin (picropodophyllin), an indolequinone (mitomycin C), a quinoline alkaloid (gimatecan), and a cyclic peptide (actinomycin D). The six cardiovascular agents consist of four dihydropyridines (benidipine, cilnidipine, lercanidipine, and nicardipine), a benzazepine (benazepril), and a hydrazine (levosimendan). The eight anthelmintic drugs include three benzimidazoles (albendazole, mebendazole, and oxibendazole) and five avermectins (abamectin, doramectin, eprinomectin, ivermectin, and selamectin). Other FXR-active drugs include two synthetic steroids (ethinylestradiol and ethylestrenol), an anticoagulant pyrimidine (dipyridamole), an antinematodal isoquinoline (emetine), an anti-infective aminoacridine (ethacridine lactate), an anti-gout alkaloid (colchicine), and a pyrazole drug to treat nicotine addiction (surinabant).

There are six target classes of these FXR-active drugs targeting DNA, tubulin, calcium channel, enzymes, nuclear receptors, and other miscellaneous targets (Figure 4b). The compounds directly binding tubulin are four benzimidazoles (the three anthelmintics plus nocodazole)^30^, three vinca alkaloids (vinblastine, vincristine, and vinorelbine)^31^, and colchicine^32^. These tubulin binders partially inhibited CDCA-induced FXR transactivation in a concentration-dependent manner (Figure 4c/d and Table S3). Other tubulin binders including a benzofuran (griseofulvin)^33^ and a taxane (docetaxel)^34^ also exhibited antagonistic effects on CDCA-induced FXR-*bla* transactivation (Table S1). Five anthracyclines (daunorubicin, doxorubicin, epirubicin, idarubicin, and nemorubicin)^35^, an aminoacridine (ethacridine lactate)^36^, and a cyclic peptide (actinomycin D)^37^ are DNA binders. Mitomycin C, an indolequinone, is a DNA crosslinker^38^. Gimatecan, a quinolone alkaloid, is a topoisomerase I inhibitor^39^. FXR-active compounds that modulate calcium channels include four dihydropyridine-based calcium channel blockers^40^ and a calcium sensitizer (levosimendan)^41^. Benazpril, dipyridamole, and flavopiridol are enzyme inhibitors targeting angiotensin-converting-enzyme (ACE)^42^, phosphodiesterase (PDE)^43^, and cyclin-dependent kinase (CDK)^44^, respectively. Picropodophylin and surinabant binds insulin-like growth factor 1 receptor (1GF1R)^45^ and cannabinoid receptor type 1 (CB_1_)^46^, respectively. Avermectin derivatives (abamectin, doramectin, eprinomectin B1a, ivermectin, and selamectin) are agonists of the gamma-aminobutyric acid (GABA) receptor^47^. The synthetic steroids are used as an ER agonist (ethinylestradiol)^48^ or as an AR agonist (ethylestrenol)^49^. Emetine is reported to bind the 40S subunit of ribosome^50^.

### Selectivity of FXR-active compounds against a family of nuclear receptors

**Figure 5:**
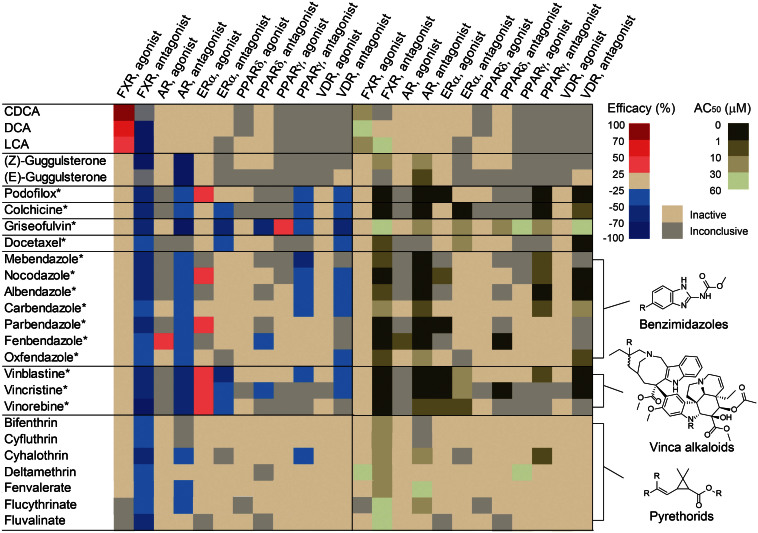
Selectivity of FXR actives in a group of nuclear receptors. The six nuclear receptor assays based on the β-lactamase reporter technology were conducted in both agonist and antagonist modes. * Denotes tubulin binders. AR, Androgen receptor; ERα, Estrogen receptor alpha; FXR, Farnesoid X receptor; PPARδ, Peroxisome proliferator-activated receptor delta; PPARγ, Peroxisome proliferator-activated receptor gamma; VDR, Vitamin D receptor.

To detect potential assay artifacts and study compound selectivity, activity patterns of the top twenty-seven FXR-active clusters against AR, ERα, PPARδ, PPARγ, and VDR, which used the same β-lactamase reporter gene technology (Table S4 and Table S5), were evaluated. Retinoic acids (k20.7) and alpha-cyano (type II) pyrethroids (k28.24) were found to be the top clusters enriched in FXR selective agonists and FXR selective antagonists, respectively (Figure S1 and Figure 5). Unlike k28.24, the noncyano (type I) pyrethroid (k28.23) were two-fold more responsive to PPARγ than FXR (Table S5). The albendazole-like benzimidazoles (k7.9) and the nocodazole-like benzimidazoles (k7.10) shared similar selectivity patterns in the AR-*bla* antagonist and the FXR-*bla* antagonist screening (Figure 5). The nicardipine-like dihydropyridine cluster (k10.12) showed antagonistic or cytotoxic response in all tested β-lactamase assays for nuclear receptors (Figure S2). Benidipine, manidipine, and nicardipine exhibited weak FXR agonist activity. The nifedipine-like dihydropyridine cluster (k11.12) yielded several distinct selectivity profiles. Felodipine, lacidipine, and lemildipine activated AR, ER, FXR, and PPARδ. Cilnidipine and nitrendipine were found to be FXR agonists, and nitrendipine was also an AR antagonist. Nimodipine, nifedipine, and nisoldipine were identified as antagonists of FXR and AR. The cholic acids (CDCA, DCA, and LCA; Figure 5) and the FXR-active benzoic retinoic acids (AM80 and AM580; Figure S1) selectively induced transactivation of FXR. Guggulsterones exhibited antagonist activity to AR and FXR (Figure 5). Some FXR-active pyrethroid insecticides such as cyfluthrin and bifenthrin were highly selective FXR antagonists (Figure 5). The anthracycline chemotherapeutics, including doxorubicin, daunorubicin, idarubicin, and pirarubicin, displayed mixed agonist/antagonist response against AR, ERα, and FXR, and antagonist response against PPARγ, PPARδ, and VDR (Figure S3). The six structural classes of tubulin binders showed distinct selectivity patterns (Figure 5). Griseofulvin, colchicine, podofilox, and the majority of FXR-active benzimidazoles (i.e., nocodazole, carbendazole, and mebendazole) shared similar activity patterns in inhibiting β-lactamase expression driven by FXR, AR, PPARγ, and VDR. Griseofulvin and colchicine also antagonized estradiol-induced ER transactivation. Three FXR-active vinca alkaloid-based tubulin binders acted as mixed agonists/antagonists of ER and antagonists of AR and FXR (Figure 5).

## Discussion

In the present study, we used a qHTS platform in combination with chemoinformatics to profile 8,599 unique environmental chemicals and drugs for their potential to modulate FXR signaling. False positive and false negative rates were minimized by using a format consisting of three replicate concentration-response curves with 15 concentrations for each test compound in primary screening. Additionally, the FXR results were compared with Tox21 qHTS screening results against several additional targets to identify non-specific effects such as compound autofluorescence, cytotoxicity, reporter gene-dependent response, or other artifacts. Overall, the high data reproducibility of replicate compounds indicated that the screening was robust. The mismatch rates of the 10K triplicate runs and the 88 replicates were both less than 1%, indicating good assay reproducibility. From the primary screening, more FXR antagonists than FXR agonists were identified. Interestingly, many of the FXR-active drugs identified (e.g., k1.10 for anthracycline chemotherapeutics and k7.9/k7.10 for dihydropyridine anti-hypertensive drugs) showed a range of agonist activity between 13% and 70% of CDCA activity as well as antagonist activity to CDCA-induced FXR activation, a pharmacological behavior consistent with classification as a partial agonist^51^. Only a few FXR agonists showed efficacies similar to CDCA, including the topical antiseptic 9-aminoacridine^36^ and the teratogenic plant-derived steroid cyclopamine^52^. About 1270 unique compounds from the Tox21 10K compound collection had inhibitory effects on CDCA-induced FXR activity. More than 542 unique of these active compounds appeared to be cytotoxic as detected by viability assays in the same well, thus requiring cluster analysis for chemical prioritization and orthogonal assays for further confirmation.

The FXR-*bla* assay is able to detect both weak and potent FXR agonists. UDCA, a 7-beta isomer of CDCA previously reported as a weak FXR agonist^5^ or a non-FXR ligand^53^, exhibited partial agonist activity at high micromolar concentrations in the FXR-*bla* assay (Figure 2a and Table S1). The potency of the other known FXR agonist GW4064 in the FXR-*bla* assay (Figure 2b and Table S1) is comparable (<5-fold difference in EC_50_ values) to the literature value measured in a coactivator recruitment assay^13^. The positive control CDCA was the most efficacious FXR agonist compared to the other cholic acids, DCA and LCA, which showed weak agonistic effects (Figure 2a) and cytotoxicity at high concentrations (Table S1). The induction of FXR activity by these cholic acids is highly specific. It is likely due to the fact that cholic acids have a shape distinct from other endogenous steroids and the ligand binding domain of FXR adopts a unique orientation for binding of cholic acids^54^. Both (*E*) and (*Z*) isoforms of guggulsterone were confirmed as FXR antagonists in both the primary screening and confirmatory assays. (*Z*)-guggulsterone was used as the antagonist control in the FXR screening and its (*E*) isoform also showed antagonist response in the AR-*bla* assay. Although guggulsterones were first discovered as naturally occurring FXR antagonists, they also bind other steroid receptors including the glucocorticoid receptor, the mineralocorticoid receptor, and the progesterone receptor^55^, making it complicated to interpret the effects of guggulsterones in FXR signaling and lipid metabolism^8^.

The recently discovered FXR agonist ivermectin^10^ and the other five avermectin macrolide antiparasitic agents (k3.3) identified in this study potently induced FXR-*bla* transactivation at submicromolar concentrations (Table S1). Ivermectin not only showed partial agonist activity but also exhibited antagonistic activity in the FXR-*bla* assay. This observation is consistent with an independent study in which ivermectin was identified in both biochemical and cell-based assays as a potent FXR antagonist^56^. Ivermectin, an avermectin antiparasitic drug used in humans, reduces hyperglycemia and hyperlipidemia symptoms in the diabetic mice model at submicromolar concentrations via FXR-mediated signaling^10^. Human patients with overdoses of ivermectin, abamectin, and emamectin have shown acute cardiotoxicity, acute neurotoxicity, or adverse effects on the gastrointestinal tract^57^ although linkage to FXR-mediated effect is not known. Doramectin, an ivermectin analog for anti-parasitic treatment in dogs, was two-fold more potent and more efficacious than ivermectin in activating FXR in our screening. In addition, the two milbemycin macrolides—milbemectin and moxidectin which lack the disaccharide moiety present in avermectins exhibited weak and no activity in the FXR-*bla* assay (Table S1). These results indicate the importance of the disaccharide in forming hydrogen bonds with FXR as observed in the co-crystal structure of ivermectin-bound FXR-LBD^10^.

We identified 63% (12 of 19) of the tested dihydropyridine-class calcium channel blockers as FXR agonists or FXR antagonists with low to high micromolar potencies (Table S1 and Figure S2). Two recently reported FXR antagonists, nimodipine and felodipine^56^, were identified as an antagonist and an agonist in the FXR-*bla* assay, respectively. The dihydropyridine drugs in the Tox21 10K compound collection were grouped into two clusters represented by nicardipine (k10.12) and nifedipine (k11.12). The two clusters exhibited distinct FXR activity and selectivity towards the other five nuclear receptors (Figure S2). The major structural difference of the two clusters is the extra phenyl group in the nicardipine-like compounds which may introduce additional steric hindrance and hydrophobic interactions in binding of FXR and other nuclear receptors. When compared with other functionally related receptors such as the pregnane X receptor (PXR), the majority of the FXR-active dihydropyridines are more potent in activating PXR^58^. Accidents of toddlers over-dosed with nifedipine, a dihydropyridine drug used to treat hypertension and to control angina, resulted in hypotension, lethargy, and/or vomiting^59^. Adults overdosed with nifedipine showed symptoms such as hypotension, sinus tachycardia, and/or sinoatrial and atrioventricular nodal depression^60^. Although therapeutic doses of dihydropyridine drugs normally yield submicromolar serum concentrations^61^ and no direct link between FXR and overdose of dihydropyridines has been reported, potential impacts of dihydropyridines on FXR-related physiological function need further evaluation as cases of overdose of these drugs increase.

Sixty-seven percent (12 of 17) of the tested pyrethroid insecticides were identified as potential FXR-specific antagonists (Table S1 and Figure 5). These pyrethroids inhibited CDCA-induced FXR transactivation at high micromolar concentrations. Pyrethroid insecticides kill insects by over-stimulating the voltage-gated sodium channels^62^. Humans are exposed by pyrethroids through ingestion or occupational exposure, and can result in paresthesia^63^, gastrointestinal irritation^63^, or abnormal glucose regulation^64^. However, human exposure of pyrethroids would likely have to reach very high levels to trigger FXR effects, doses at which other modes of activity/toxicity would likely already have been manifested. One interesting finding for pyrethroids is a relationship between structure and FXR activity. The alphacyano pyrethroids (k28.24) were more enriched in FXR antagonists and more FXR-specific than the noncyano pyrethroids (k28.23) (Table S5). The main structural difference between the two clusters is the substitution of vinylic hydrogen to a cyano group in the k28.24 pyrethroids. While the majority of k28.24 pyrethroids exhibited FXR-specific antagonistic effect, cyhalothrin, fenvalerate and flucythrinate, also showed antagonist activity in other nuclear receptor assays including the AR-*bla* and the VDR-*bla* assays (Figure 5). These three compounds have an aryl group instead of the acid moiety present in most pyrethroids. These data suggest that FXR could be a novel mammalian target of pyrethroids, and that the structural differences of the FXR-active pyrethroids play a crucial role in compound activity and selectivity.

Our data provides interesting evidence that chemicals known to inhibit tubulin are active as FXR antagonists. All tested tubulin inhibitors including benzimidazoles (e.g., nocodazole), colchicine, docetaxel, griseofulvin, podofilox, and vinca alkaloids (vinblastine, vincristine, and vinorelbine) exhibited FXR antagonist activity at submicromolar to low micromolar concentrations in the FXR-*bla* assay screening (Figure 5). Despite the fact that most of these FXR-active tubulin inhibitors are mitotic inhibitors, concentration-dependent inhibitory effects of these compounds in the FXR-*bla* assay were only observed in the antagonist screening. The FXR-active benzimidazoles, vinca alkaloids, colchicine, and podofilox are 5 to 100-fold more potent in inhibiting CDCA-induced FXR activity than activating PXR^58^. Benzimidazoles have been identified as FXR agonists with lipid lowering effects *in vivo*^65^. However, the FXR-active benzimidazoles identified from the screen were unable to induce FXR-*bla* transactivation and to alter FXR coactivator recruitment (Table 1). Compared to the reported benzimidazole-based FXR agonists, the nocodazole-like benzimidazoles lack an alkylated N3 functionality important for FXR binding. Therefore these benzimidazoles are likely to modulate FXR signaling via alternative mechanisms. Tubulin inhibitors and microtubules have been implicated in regulating a number of nuclear receptors including AR^66^, the glucocorticoid receptor (GR)^67^, PXR^68^, and the retinoic acid receptor (RAR)^69^ as well as the aryl hydrocarbon receptor (AhR)^70^. Those studies suggest that microtubule inhibitors affect nuclear receptor trafficking by not allowing coregulators to move from the cytoplasm to the nucleus. Additionally, ERα was reported to activate histone deacetylase 6 (HDAC6) to deacetylate tubulins in human breast cancer MCF-7 cells^71^. Future work should interrogate the role of microtubules in FXR signaling and evaluate effects of tubulin inhibitors on FXR target genes to determine if these compounds are acting directly or indirectly on FXR activity.

In summary, the present study identified several key scaffolds of FXR-active drugs and environmental chemicals. Because FXR regulates diverse metabolic pathways, it has emerged as an important drug target and a potential toxicity mediator. Complete structure-activity relationships of anthracyclines, avermectins, dihydropyridines, and pyrethroids require further testing of a larger number of tailor-designed analogs to identify the pharmacophore important for FXR binding. Systematic evaluation of compound selectivity of FXR-active environmental chemicals against other functionally related receptors like PXR, liver X receptor (LXR), and constitutive androstane receptor (CAR)^72^, and G protein-coupled bile acid receptor (GPBAR1)^73^ is of great importance to understand how these compounds affect lipid and xenobiotic metabolism.

## Methods

### Compound library

The Tox21 10K compound library (http://www.epa.gov/ncct/dsstox/sdf_tox21s.html) is provided by the National Toxicology Program (NTP), the Environmental Protection Agency (EPA), and the NIH Chemical Genomics Center/National Center for Advancing Translational Sciences^74^. Each compound was prepared as previously described^75^ and serially diluted in DMSO in 1536-well microplates to yield 15 concentrations generally ranging from 1.1 nM to 92 μM (final concentrations in the assay wells).

### Cell culture

The cell line and the cell culture reagents were purchased from the Life Technologies (Carlsbad, CA, USA). The GeneBLAzer® FXR-UAS-bla HEK293T cells stably expressing an FXR-driven β-lactamase reporter gene were cultured in high glucose Dulbecco's Modified Eagle Medium (DMEM, Cat. No. 10569-010) supplemented with 25 mM 4-(2-hydroxyethyl)-1-piperazineethanesulfonic acid (HEPES, pH 7.3), 0.1 mM non-essential amino acids (NEAA), 100 μg/mL hygromycin, 100 μg/mL zeocin, 100 U/mL penicillin, 100 mg/mL streptomycin, and 10% dialyzed fetal bovine serum (FBS). Cells were grown at 37°C/5% CO_2_ in a humidified incubator and passaged at 70–80% confluency. To prepare FXR-UAS-bla cells for assays, cells were washed with Dulbecco's phosphate-buffered saline (DPBS), detached with 0.05% Trypsin/EDTA, and re-suspended in phenol red-free DMEM containing 1 mM sodium pyruvate, 0.1 mM NEAA, 100 U/mL penicillin, 100 mg/mL streptomycin, and 2% charcoal-stripped FBS.

### qHTS of beta-lactamase reporter gene and cell viability assays

The online screening procedures to profile the Tox21 compound collection against androgen receptors (AR), estrogen receptor alpha (ERα), farnesoid X receptor (FXR), peroxisome proliferator-activated receptor delta and gamma (PPARδ/γ), and vitamin D receptor (VDR) were adapted from the previously reported procedures^28^ and the results were deposited to the PubChem BioAssay database (Table S4). The detailed protocols of the FXR-*bla* and viability screening on the Tox21 compound collection are described as follows (Table S6 and Table S7). Five μL of suspended FXR-*bla* cells at a cell density of 1000 cells/μL was plated in 1536-well tissue culture-treated black clear bottom plates (Greiner Bio One North America, Monroe, NC, USA) using a 8-tip multidrop reagent dispenser (Thermo Fisher Scientific, Waltham, MA, USA). The standard procedure to screen FXR agonists starts with cell incubation at 37°C/5% CO_2_ for 5 hours, followed by addition of 23 nL of compound solution on a compound transfer workstation (Kalypsys, San Diego, CA, USA). Chenodeoxycholic acid (CDCA) and DMSO (Sigma-Aldrich Corp., St. Louis, MO, USA) were used as positive and negative agonist-mode controls, respectively. To screen compounds that antagonize CDCA-induced transactivation of FXR, an extra 1 μL of CDCA was added on the top of the cell/compound mixtures to achieve a final agonist concentration of 50 μM. After 16 hours of incubation at 37°C/5% CO_2_, 1 μL of CCF_4_-AM substrate reagents was added to each well using a Bioraptr Flying Reagent Dispenser (FRD) workstation (Beckman Coulter, Indianapolis, IN, USA), followed by a 2 hour incubation at room temperature in the dark. Samples were excited at 405 nm and the resulting fluorescence emission intensity values at 460 nm and 530 nm were recorded on an EnVision plate reader (Perkin Elmer, Shelton, CT, USA). The cytotoxicity effects were measured in the same plates by adding 3 μL of CellTiter Glo reagent (Promega, Madison, WI, USA) to each well and incubating the plates at room temperature in the dark for 30 min. The luminescence values were acquired on a ViewLux plate reader (Perkin Elmer, Shelton, CT, USA). The confirmatory screening used two 1536-well compound plates for 266 selected compounds, where the original stock solutions (10 mM or 20 mM) used for primary screening were serially diluted to yield eight concentrations at 1:4 dilution ratios and each dilution series was plated to the same compound plate. The same assay protocols of FXR-*bla* and viability assays in the primary screening were used to test the 266 compounds in three independent experiments, in both agonist and antagonist modes, and on an off-line screening platform.

### qHTS data analysis

The qHTS data was analyzed according to the previous protocol^28^. Briefly, raw plate reads for each titration point were first normalized relative to the positive control compound (agonist mode: CDCA, 100%; antagonist mode: guggulsterone, −100%; cell viability: tetra-n-octylammonium bromide (TOAB), −100%) and DMSO-only wells (0%) as follows: % Activity = [(V_compound_ − V_DMSO_)/(V_pos_ − V_DMSO_)] × 100, where V_compound_ denotes the compound well values, V_pos_ denotes the median value of the positive control wells, and V_DMSO_ denotes the median values of the DMSO-only wells, The data set was then corrected using the DMSO-only compound plates at the beginning and end of the compound plate stack by applying an in-house pattern correction algorithm. The half maximum activity values (AC_50_) and maximum response (efficacy) values were obtained by fitting the concentration-response curves of each compound to a four-parameter Hill equation^76^. Compounds designated as Class 1–4 according to the type of concentration–response curve observed (1.1, 1.2, 1.3, 1.4, 2.1, 2.2, 2.3, 2.4, and 3 for activators; −1.1, −1.2, −1.3, −1.4, −2.1, −2.2, −2.3, −2.4, and −3 for inhibitors; 4 for inactive) were converted to curve ranks (1 to 9 integers for increasing activating abilities; −9 to −1 integers for decreasing inhibitory abilities; 0 for inactive) according to the criteria previously described^28^. The activity outcome of a test compound in each readout was first categorized based on the average curve rank from the triplicate runs and the reproducibility calls. The final activity outcome of each compound was determined based on its multi-channel readout activity, for example, compounds with inactive curve ranks in both the 460 nm channel and/or the FRET ratios were concluded to be inactive. A compound was assigned as autofluorescent and inconclusive when the efficacy at 460 nm is two-fold greater than the efficacy of the ratiometric readout. Compounds that antagonize FXR and kill cells at similar potencies (i.e., when AC_50, viability_/AC_50, ratio_ < 3, p < 0.05) were considered as cytotoxic and inconclusive. Data reproducibility was categorized as active match, inactive match, inconclusive, and mismatch according to the previously described criterion based on activity outcome differences between replicates and percentage of inactive outcomes^28^,^77^. The 10K library was clustered based on structural similarity (Leadscope® fingerprints; Leadscope, Inc., Columbus, OH, USA) using the self-organizing map (SOM) algorithm^29^. Each cluster was evaluated for its enrichment of active agonists/antagonists and significance of enrichment was determined by p-values from the Fisher's exact test.

### FXR coactivator recruitment assay

The glutathione S-transferase (GST)-tagged FXR-LBD protein, terbium (Tb)-labeled goat anti-GST antibody, and fluorescein-labeled SRC2-2 coactivator peptide were purchased from the Life Technologies (Carlsbad, CA, USA). The ability of an FXR agonist to recruit a coactivator is reported by FRET between the donor Tb-labeled antibody (λ_ex_ = 340 nm and λ_em_ = 495 nm) and the acceptor fluorescein-labeled peptide (λ_em_ = 520 nm), where the agonist induces conformation changes of FXR-LBD and leads to coactivator recruitment. Six μL of 5 nM GST-tagged FXR-LBD, 0.1% bovine serum albumin (Sigma-Aldrich, St. Louis, MO, USA), 5 nM Tb-labeled anti-GST antibody, and 500 nM fluorescein-labeled SRC2-2 were plated in a 1536-well black solid bottom plate (Greiner Bio One North America, Monroe, NC, USA) using a Mantis single-channel liquid dispenser (Formulatrix, Inc., Waltham, MA, USA). Twenty-three nL of compound solution in eight concentrations in DMSO was added to the corresponding well using a compound transfer workstation (Wako Automation, San Diego, CA, USA). The reaction was incubated in the dark at room temperature for 30 minutes. Samples were excited at 340 nm, and the resulting emission intensity values at 495 nm and 520 nm with 90 μs delay time and 300 μs integration time were acquired on the EnVision plate reader (Perkin Elmer, Shelton, CT, USA). The antagonist assay was conducted in the presence of 50 μM CDCA to induce coactivator recruitment. The TR-FRET ratios were normalized using DMSO as 0% FXR activity and 50 μM CDCA as 100% FXR activity for both agonist and antagonist mode screening.

## Supplementary Material

Supplementary Information Supplemental information

## Figures and Tables

**Table 1:** Compound potency (μM, EC_50_/IC_50_) and efficacy (% in parenthesis) in FXR-*bla*, viability, and TR-FRET co-activator assays

Compound Name (CAS No.)	Chemical Structure	Cluster No.	FXR-bla, agonist EC_50_, μM (Efficacy, %)	FXR-bla, antagonist IC_50_, μM (Efficacy, %)	Cell viability IC_50_, μM (Efficacy, %)	FXR TR-FRET, agonist EC_50_, μM (Efficacy, %)	FXR TR-FRET, antagonist IC_50_, μM (Efficacy, %)
Albendazole (54965-21-8)		k7.9	Inactive	0.37 ± 0.07 (52 ± 2)	Inactive	Inactive	Inactive
9-Aminoacridine (52417-2208)		k31.8	11.17 ± 2.09 (152 ± 22)	Inactive	Inactive	Inactive	Inactive
Actinomycin D (50-76-0)		k10.3	Inactive	0.02 ± 0.01 (77 ± 27)	Inactive	Inactive	Inactive
Colchicine (64-86-8)		k2.20	Inactive	0.03 ± 0.01 (54 ± 6)	Inactive	Inactive	0.03 ± 0.01 (52 ± 16)
Cyclopamine (4449-51-8)		k4.4	10.57 ± 3.13 (94 ± 9)	Inactive	17.56 ± 6.39 (51 ± 14)	Inactive	Inactive
Daunorubicin (20830-81-3)		k1.10	1.02 ± 0.33 (48 ± 14)	5.53 ± 0.00 (105 ± 1)	Inactive	Inactive	5.55 ± 0.64 (221 ± 43)
Diphacinone (82-66-6)		k18.15	Inactive	15.02 ± 0.98 (87 ± 0)	Inactive	Inactive	Inactive
Dipyridamole (58-32-2)		k6.5	Inactive	3.50 ± 0.40 (81 ± 5)	Inactive	Inactive	Inactive
Doxorubicin (25316-40-9)		k1.10	1.35 ± 0.00 (68 ± 8)	2.80 ± 0.19 (114 ± 9)	Inactive	Inactive	4.39 ± 2.32 (185 ± 54)
Emetine (316-42-7)		k2.24	Inactive	4.23 ± 0.28 (120 ± 19)	Inactive	Inactive	Inactive
Enzastaurin (170364-57-5)		k38.3	Inactive	10.23 ± 0.69 (110 ± 9)	Inactive	Inactive	Inactive
Epirubicin (56390-09-1)		k1.10	5.78 ± 0.74 (44 ± 7)	17.80 ± 4.07 (108 ± 1)	Inactive	Inactive	7.91 ± 1.48 (233 ± 15)
Flavopiridol (146426-40-6)		k1.12	Inactive	0.02 ± 0.00 (123 ± 7)	Inactive	Inactive	Inactive
Gimatecan (292618-32-7)		k1.7	Inactive	2.69 ± 1.07 (87 ± 9)	Inactive	Inactive	Inactive
Idarubicin (57852-57-0)		k1.10	1.37 ± 0.38 (42 ± 16)	4.07 ± 0.28 (107 ± 3)	Inactive	Inactive	6.29 ± 1.32 (162 ± 41)
Mebendazole (31431-39-7)		k7.10	Inactive	2.19 ± 1.51 (70 ± 9)	Inactive	Inactive	Inactive
Nemorubicin (108852-90-0)		k1.10	Inactive	0.13 ± 0.01 (99 ± 6)	Inactive	Inactive	5.75 ± 0.39 (191 ± 23)
Nocodazole (31430-18-9)		k7.10	Inactive	0.29 ± 0.19 (68 ± 20)	Inactive	Inactive	Inactive
Oxibendazole (20559-55-1)		k5.20	Inactive	0.58 ± 0.07 (55 ± 5)	Inactive	Inactive	Inactive
Picropodophyllin (518-28-5)		k3.21	Inactive	0.02 ± 0.00 (55 ± 3)	10.72 ± 14.88 (42 ± 14)	Inactive	Inactive
Proflavin hemisulfate (1811-28-5)		k30.7	Inactive	9.39 ± 3.90 (142 ± 12)	Inactive	2.21 ± 0.25 (182 ± 5)	Inactive
Surinabant (288104-79-0)		k24.3	Inactive	1.84 ± 0.77 (86 ± 25)	17.14 ± 1.12 (71 ± 5)	Inactive	0.20 ± 0.03 (73 ± 1)
Vinblastine (143-67-9)		k2.26	Inactive	0.10 ± 0.04 (57 ± 7)	Inactive	Inactive	Inactive
Vincristine (2068-78-2)		k2.26	Inactive	0.12 ± 0.02 (61 ± 11)	12.62 ± 10.47 (45 ± 7)	Inactive	Inactive
Vinorelbine (125317-39-7)		k2.26	Inactive	0.03 ± 0.01 (62 ± 1)	Inactive	Inactive	Inactive

Compound IC_50_/EC_50_ value (concentration of half maximal inhibition or activation) and efficacy (inhibition or activation as percent of positive control) are the mean ± SD of the results from 3 experiments.
