# Supplementary material for: Quantitative High-Throughput Profiling of Environmental Chemicals and Drugs that Modulate Farnesoid X Receptor
Source: Sci Rep. 2014 Sep 26;4:6437. doi: 10.1038/srep06437 (PMC4894417; doi:10.1038/srep06437)
Supplement: Supplementary Information — Supplemental information [file srep06437-s1.pdf]

**Supplementary Materials**

Quantitative High Throughput Profiling of Environmental Chemicals and Drugs that Modulate Farnesoid X Receptor

Chia-Wen Hsu[1], Jinghua Zhao[1], Ruili Huang[1], Jui-Hua Hsieh[2], Jon Hamm[3], Xiaoqing Chang[3], Keith Houck[4], Menghang Xia[1]*

[1]National Center for Advancing Translational Sciences, National Institutes of Health, Bethesda, MD, USA

[2]Division of the National Toxicology Program and National Institute of Environmental Health Sciences, National Institutes of Health, Research Triangle Park, NC, USA

[3]Integrated Laboratory Systems, Inc., Morrisville, NC, USA

[4]U.S. Environmental Protection Agency, Research Triangle Park, NC, USA

*Address correspondence to:

Menghang Xia, Ph.D.

National Institutes of Health

National Center for Advancing Translational Sciences

9800 Medical Center Drive

Bethesda, MD 20892

Phone: 301-217-5718

Email: mxia@mail.nih.gov

**Table S1**. Assay data and description of identified FXR-active compounds in the order of cluster number.

| Compound Name (CAS No.) | Chemical Structure | $EC_{50}/IC_{50}$, μM (Efficacy, %) | | | Cluster # |
|---|---|---|---|---|---|
| | | FXR agonist mode | FXR antagonist mode | Cytotoxicity | |
| Pirarubicin (72496-41-4) | 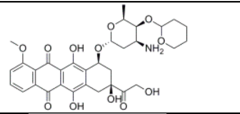 | 16.91 ± 10.98 (20 ± 9) | 21.28 ± 15.56 (60 ± 23) | Inactive | k1.10 |
| Biochanin A (491-80-5) | 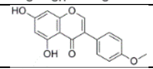 | Inactive | 12.07 ± 3.02 (62 ± 10) | Inactive | k1.13 |
| Genistein (446-72-0) | 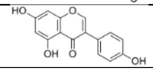 | Inactive | 22.95 ± 3.16 (57 ± 6) | Inactive | k1.13 |
| Digoxin (20830-75-5) | 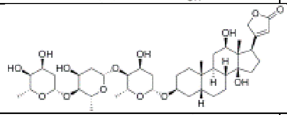 | Inactive | 0.08 ± 0.02 (92 ± 10) | 0.02 ± 0.01 (35 ± 7) | k2.2 |
| Apigenin (520-36-5) | 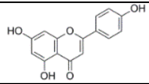 | nactive | 18.74 ± 6.79 (88 ± 30) | Inactive | k2.12 |
| Chrysin (480-40-0) | 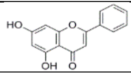 | Inactive | 46.19 ± 10.42 (109 ± 27) | Inactive | k2.12 |
| Griseofulvin (126-07-8) | 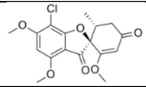 | Inactive | 42.35 ± 17.31 (42 ± 7) | Inactive | k2.15 |
| Abamectin (71751-41-2) | 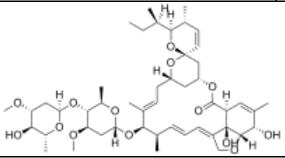 | 0.78 ± 0.09 (33 ± 4) | 3.77 ± 0.25 (127 ± 3) | 5.12 ± 0.35 (104 ± 3) | k3.3 |
| Doramectin (117704-25-3) | 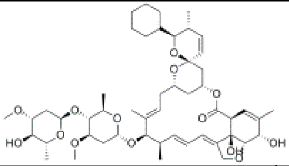 | 0.44 ± 0.16 (62 ± 9) | 18.20 ± 1.23 (152 ± 10) | 11.03 ± 0.00 (116 ± 6) | k3.3 |
| Emamectin (155569-91-8) | 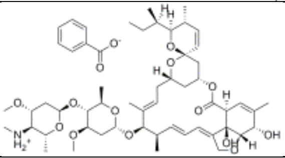 | 24.64 ± 8.97 (56 ± 26) | 5.81 ± 2.68 (64 ± 19) | 7.54 ± 1.29 (116 ± 14) | k3.3 |
| Eprinomectin B1a (133305-88-1) | 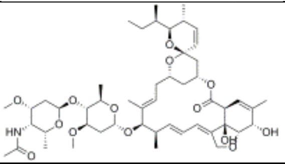 | 5.33 ± 0.35 (41 ± 1) | 14.46 ± 0.98 (110 ± 6) | 13.95 ± 1.60 (108 ± 4) | k3.3 |
| Eprinomectin B1a, B1b mixture (1142337-10-7) | 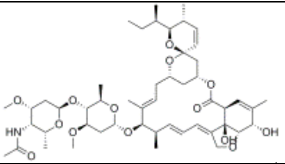 | 1.00 ± 0.09 (34 ± 4) | 3.77 ± 0.25 (117 ± 4) | 4.07 ± 0.28 (106 ± 5) | k3.3 |
| Ivermectin B1a* (71827-03-7) | 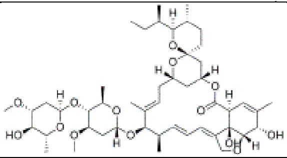 | 0.57 ± 0.18 (27 ± 2) | 2.50 ± 0.52 (120 ± 8) | 14.07 ± 2.63 (124 ± 6) | k3.3 |

| | | | | | |
|---|---|---|---|---|---|
| Milbemectin (mixture of >=70% Milbemcin A4 & <= 30% Milbemycin A4 (NOCAS_34742) | 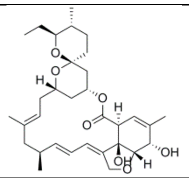 | 37.71 ± 15.34 (90 ± 46) | 11.80 ± 18.92 (74 ± 23) | 13.03 ± 3.84 (08 ± 7) | k3.3 |
| Selamectin (165108-07-6) | 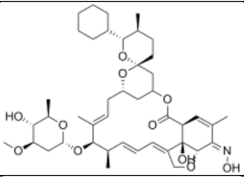 | 5.98 ± 0.39 (68 ± 5) | 2.48 ± 0.82 (45 ± 6) | Inactive | k3.3 |
| Docetaxel (114977-28-5) | 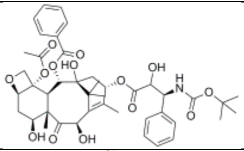 | Inactive | 0.008 ± 0.005 (35 ± 4) | Inactive | k3.10 |
| Podofilox (518-28-5) | 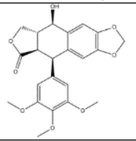 | Inactive | 0.018 ± 0.006 (54 ± 2) | Inactive | k3.21 |
| Albendazole oxide (54029-12-8) | 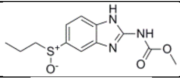 | Inactive | 27.93 ± 5.02 (35 ± 16) | Inactive | k7.9 |
| Benomyl (17804-35-2) | 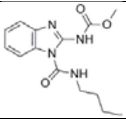 | Inactive | 18.28 ± 3.32 (21 ± 18) | Inactive | k7.9 |
| Carbendazole (10605-21-7) | 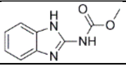 | Inactive | 12.26 ± 2.90 (36 ± 12) | Inactive | k7.9 |
| Parbendazole (14255-87-9) | 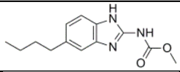 | Inactive | 0.16 ± 0.14 (43 ± 3) | Inactive | k7.9 |
| Cyclobendazole (31431-43-3) | 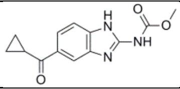 | Inactive | 3.00 ± 1.57 (44 ± 2) | Inactive | k7.10 |
| Fenbendazole (43210-67-9) | 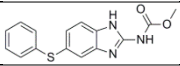 | Inactive | 0.49 ± 0.26 (30 ± 2) | Inactive | k7.10 |
| Oxfendazole (53716-50-0) | 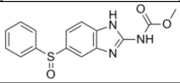 | Inactive | 7.59 ± 9.91 (33 ± 3) | Inactive | k7.10 |
| Benidipine (91599-74-5) | 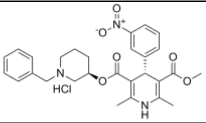 | Inactive | 24.78 ± 3.16 (84 ± 17) | Inactive | k10.12 |
| Lercanidipine (132866-11-6) | 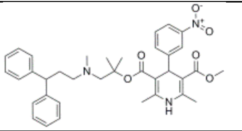 | Inactive | 15.54 ± 9.59 (-71 ± 10) | Inactive | k10.12 |
| Nicardipine (54527-84-3) | 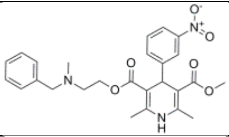 | 3.64 ± 0.84 (33 ± 7) | 16.65 ± 5.67 (116 ± 10) | 31.86 ± 15.85 (125 ± 27) | k10.12 |
| Manidipine (89226-50-6) | 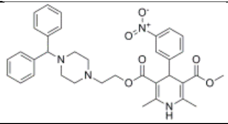 | 6.92 ± 4.00 (13 ± 1) | 19.24 ± 10.41 (57 ± 35) | 18.25 ± 15.09 (49 ± 17) | k11.12 |

| | | | | | |
|---|---|---|---|---|---|
| Cilnidipine (132203-70-4) | 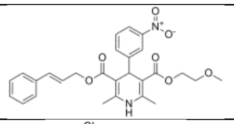 | 8.08 ± 3.40 (64 ± 7) | 31.06 ± 2.10 (64 ± 12) | Inactive | k11.12 |
| Felodipine* (72509-76-3) | 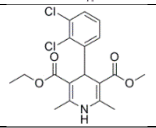 | 28.77 ± 1.87 (30 ± 9) | Inactive | Inactive | k11.12 |
| Lacidipine (103890-78-4) | 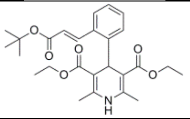 | 23.51 ± 10.08 (70 ± 41) | 32.27 ± 2.10 (55 ± 20) | 54.45 ± 39.49 (56 ± 39) | k11.12 |
| Lemildipine (94739-29-4) | 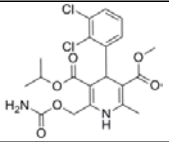 | 28.90 ± 3.98 (54 ± 26) | Inactive | Inactive | k11.12 |
| Nifedipine (21829-25-4) | 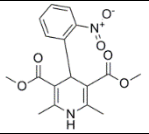 | Inactive | 34.02 ± 21.36 (66 ± 46) | Inactive | k11.12 |
| Nimodipine* (66085-59-4) | 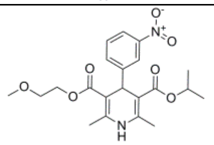 | Inactive | 31.06 ± 2.10 (55 ± 2) | Inactive | k11.12 |
| Nisoldipine (63675-72-9) | 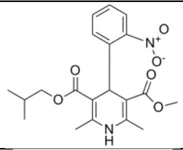 | Inactive | 32.65 ± 20.22 (73 ± 29) | Inactive | k11.12 |
| Nitrendipine (39562-70-4) | 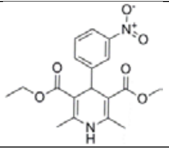 | 18.13 ± 14.04 (15 ± 5) | Inactive | Inactive | k11.12 |
| Bortezomib (179324-69-7) | 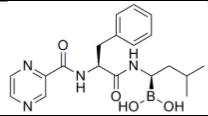 | Inactive | 0.44 ± 0.21 (101 ± 9) | 0.22 ± 0.10 (57 ± 2) | k13.4 |
| AM 80 (94497-51-5) | 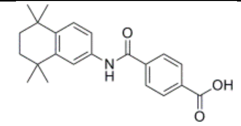 | 38.85 ± 2.36 (18 ± 7) | Inactive | Inactive | k20.7 |
| AM 580 (102121-60-8) | 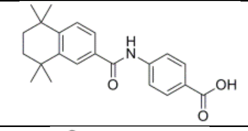 | 45.42 ± 3.07 (68 ± 37) | Inactive | Inactive | k20.7 |
| 2,2-Dithiobisbenzanilide (DTBBA) (135-57-9) | 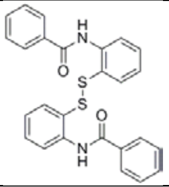 | 24.61 ± 15.30 (31 ± 13) | 18.02 ± 8.46 (51 ± 17) | 14.17 ± 8.63 (47 ± 23) | k20.7 |
| 17α-Estradiol (57-91-0) | 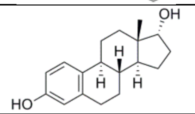 | Inactive | 31.97 ± 11.71 (79 ± 13) | Inactive | k20.11 |

| | | | | | |
|---|---|---|---|---|---|
| 17β-Estradiol (50-28-2) | 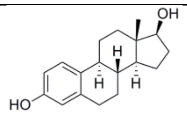 | Inactive | 33.15 ± 9.81 (94 ± 17) | Inactive | k20.11 |
| Ethinylestradiol (57-63-6) | 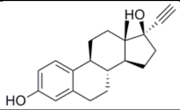 | Inactive | 16.65 ± 4.89 (91 ± 20) | 51.78 ± 4.47 (36 ± 47) | k20.11 |
| Fulvestrant (129453-61-8) | 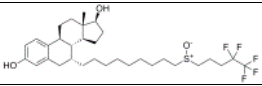 | Inactive | 13.11 ± 12.17 (50 ± 27) | Inactive | k20.11 |
| 13-*cis* Retinoic acid (4759-48-2) | 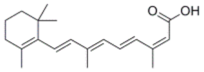 | 51.11 ± 3.46 (50 ± 16) | 0.15 ± 0.21 (41 ± 13) | 34.47 ± 29.18 (52 ± 44) | k22.22 |
| Tretinoin (302-79-4) | 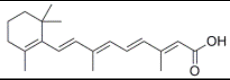 | 39.02 ± 13.80 (35 ± 15) | 14.94 ± 26.82 (43 ± 10) | Inactive | k22.22 |
| Bifenthrin (82657-04-3) | 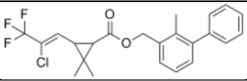 | Inactive | 11.35 ± 4.58 (50 ± 16) | Inactive | k28.23 |
| Kadethrin (58769-20-3) | 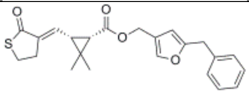 | Inactive | 9.36 ± 2.26 (40 ± 6) | Inactive | k28.23 |
| Tefluthrin (79538-32-2) | 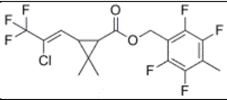 | Inactive | 12.38 ± 2.01 (46 ± 7) | Inactive | k28.23 |
| Cyfluthrin (68359-37-5) | 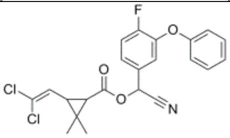 | Inactive | 21.18 ± 2.91 (43 ± 17) | Inactive | k28.24 |
| Cyhalothrin (68085-85-8) | 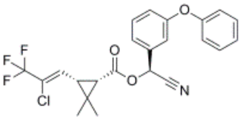 | Inactive | 21.32 ± 13.05 (50 ± 15) | Inactive | k28.24 |
| Cypermethrin (52315-07-8) | 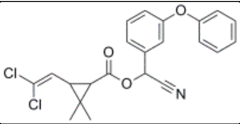 | Inactive | 33.90 ± 16.61 (36 ± 15) | Inactive | k28.24 |
| Deltamethrin (52918-63-5) | 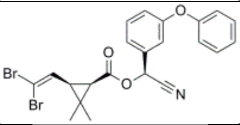 | Inactive | 32.68 ± 11.03 (47 ± 24) | Inactive | k28.24 |
| Esfenvalerate (66230-04-4) | 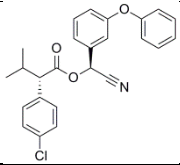 | Inactive | 13.79 ± 0.00 (34 ± 16) | Inactive | k28.24 |
| Fenvalerate (51630-58-1) | 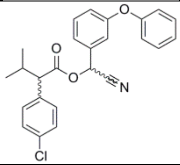 | Inactive | 23.85 ± 12.41 (38 ± 6) | Inactive | k28.24 |

| | | | | | |
|---|---|---|---|---|---|
| Flucythrinate (70124-77-5) | 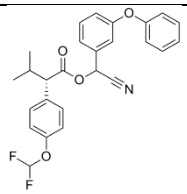 | Inactive | 31.40 ± 20.51 (37 ± 9) | Inactive | k28.24 |
| Fluvalinate (69409-94-5) | 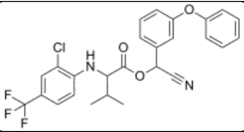 | Inactive | 30.26 ± 11.97 (53 ± 3) | Inactive | k28.24 |
| Phenothrin (26002-80-2) | 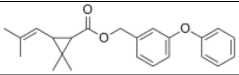 | Inactive | 25.42 ± 11.80 (27 ± 7) | Inactive | k28.24 |
| (E)-Guggulsterone* (39025-24-6) | 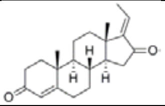 | Inactive | 24.06 ± 0.00 (58 ± 33) | Inactive | k37.26 |
| (Z)-Guggulsterone* (39025-23-5) | 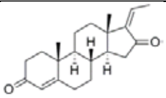 | Inactive | 39.05 ± 16.97 (124 ± 4) | Inactive | k37.26 |
| Chenodeoxycholic acid (CDCA)* (474-25-9) | 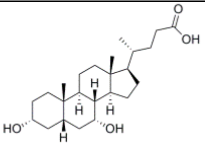 | 28.62 ± 1.55 (119 ± 35) | Inactive | 55.47 ± 8.99 (50 ± 1) | k39.24 |
| Deoxycholic acid (DCA)* (302-95-4) | 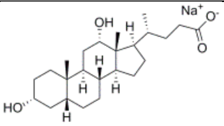 | 47.31 ± 5.84 (50 ± 12) | Inactive | 66.52 ± 16.09 (73 ± 17) | k39.24 |
| Litholic acid (LCA)* (434-13-9) | 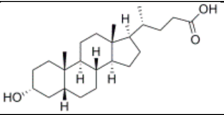 | 25.06 ± 17.19 (20 ± 7) | 51.90 ± 3.39 (75 ± 40) | 43.75 ± 4.18 (65 ± 22) | k39.24 |
| Ursodeoxycholic acid (UDCA)* (128-13-2) | 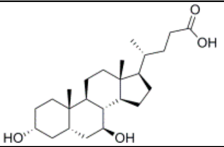 | 120.70 ± 10.41 (47 ± 3) | 0.007 ± 0.010 (20 ± 16) | Inactive | k39.24 |
| GW4064* | 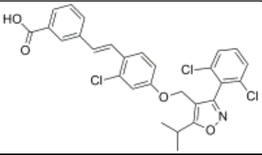 | 0.003 ± 0.001 (119 ± 11) | Inactive | Inactive | k26.1 |

* denotes for known ligands of FXR.

$EC_{50}$: concentration calculated to induce a half maximal response. $IC_{50}$: concentration calculated to inhibit a half maximal response.

**Table S2**. Representative FXR-active structural clusters identified from FXR-*bla* screens.

| Cluster # | Structural Class | Description | Example |
|---|---|---|---|
| k1.10 | Anthracyclines | Chemotherapeutic drugs. | Doxorubucin |
| k1.13 | Isoflavonoids | Phytochemicals. Antioxidants. | Genistein |
| k1.14 | Rotenones | Insecticides. | Rotenone |
| k2.12 | Flavones | Phytochemicals. | Apigenin |
| k2.20 | Colchicine | A drug to treat gout. | Colchicine |
| k2.26 | Vinca alkaloids | Phytochemicals. Anticancer drugs. | Vinblastine |
| k~~K~~3.3 | Avermectins | Antiparasitic drugs. | Ivermectin |
| k6.1 | Cytidines | Nucleoside analogs. | 5-Azacytidine |
| k7.9 | Benzimidazoles | Antifungals. Fungicides. | Albendazole |
| k7.10 | Benzimidazoles | Antineoplastic agents. Antiparasitics. | Nocodazole |
| k7.15 | Estrogens | Synthetic estrogens. | Zearalenone |
| k10.12 | Dihydropyridines | Antihypertension drugs. | Nicardipine |
| k11.12 | Dihydropyridines | Antihypertension drugs. | Nimodipine |
| k14.11 | Pyridazines | Calcium sensitizers. Enzyme inhibitors | Levosimendan |
| k18.15 | 1,3-Indandiones | Rodenticides. | Diphacinone |
| k20.11 | Estradiols | Natural and synthetic estrogens. | 17$\alpha$-Estradiol |
| k20.18 | Diphenyl ethers | Herbicides. | Lactofen |
| k20.7 | Retinobenzoic acids | RAR$\alpha$ ligands. | AM580 |
| k22.1 | Imidazoles | Antifungals. | Sulconazole |
| k22.22 | Retinoic acids | Vitamin A metabolites and analogs. | 13-cis Retinoic acid |
| k24.1 | Organochlorides | Insecticides. | p,p'-DDT |
| k28.23 | Pyrethroids | Insecticides. | Bifenthrin |
| k28.24 | Pyrethroids | Insecticides. | Cyfluthrin |
| k35.18 | Thiocarbamates | Drugs and fungicides. | Dithiurum |
| k37.26 | Pregnanes | Natural and synthetic hormones. | (Z)-guggulsterones |
| k38.14 | Organochlorines | Insecticides. | Dieldrin |
| k38.3 | Bisindolylmaleimides | Antineoplastic agents, kinase inhibitors. | Enzastaurin |
| k39.24 | Cholic acids | Primary and secondary bile acids. | Chenodeoxycholic acid |

RAR$\alpha$: retinoic acid-activated receptor alpha. DDT: dichlorodiphenyltrichloroethane.

**Table S3.** FXR-*bla* assay data of the top 30 potential FXR-active drugs with known modes of action.

| Compound Name | $EC_{50}$, μM (Efficacy, %) | | | Cluster # | Compound Description |
|---|---|---|---|---|---|
| | FXR agonist | FXR antagonist | Cytotoxicity | | |
| Abamectin (71751-41-2) | 0.78 ± 0.09 (33 ± 4) | 3.77 ± 0.25 (127 ± 3) | 5.12 ± 0.35 (104 ± 3) | k3.3 | Anthelmintic and insecticide. GABA agonist[1]. |
| Actinomycin D (50-76-0) | Inactive | 0.018 ± 0.012 (77 ± 27) | Inactive | k10.3 | Antibiotic. Anticancer agent. DNA binder[2]. |
| Albendazole (54965-21-8) | Inactive | 6.04 ± 9.33 (56 ± 40) | Inactive | k7.9 | Anthelmintic. Tubulin binder[3]. |
| Benazepril (86541-74-4) | 13.39 ± 1.54 (36 ± 5) | Inactive | 29.85 ± 0.00 (87 ± 27) | k15.1 | Cardiovascular agent. ACE inhibitor[4]. |
| Benidipine (91599-74-5) | Inactive | 24.78 ± 3.16 (84 ± 17) | Inactive | k10.12 | Cardiovascular agent. Calcium channel blocker[5]. |
| Cilnidipine (132203-70-4) | 8.08 ± 3.40 (64 ± 7) | 31.06 ± 2.10 (64 ± 12) | Inactive | k11.12 | Cardiovascular agent. Calcium channel blocker[5]. |
| Colchicine (64-86-8) | Inactive | 0.011 ± 0.015 (55 ± 11) | Inactive | k2.20 | Drug to treat gout attack. Tubulin binder[6]. |
| Daunorubicin (20830-81-3) | 2.36 ± 1.17 (93 ± 18) | 5.84 ± 2.20 (97 ± 3) | Inactive | k1.10 | Anticancer agent. DNA intercalator[7]. |
| Dipyridamole (58-32-2) | Inactive | 6.13 ± 1.71 (53 ± 11) | Inactive | k6.5 | Anticoagulant drug. PDE inhibitor[8]. |
| Doramectin (117704-25-3) | 0.44 ± 0.16 (62 ± 9) | 18.20 ± 1.23 (152 ± 10) | 11.03 ± 0.00 (116 ± 6) | k3.3 | Anthelmintic. GABA agonist[1]. |
| Doxorubicin (25316-40-9) | 0.98 ± 0.50 (32 ± 7) | 2.74 ± 0.59 (98 ± 6) | Inactive | k1.10 | Anticancer agent. DNA intercalator[7]. |
| Emetine (316-42-7) | Inactive | 5.57 ± 1.00 (113 ± 6) | Inactive | k2.24 | Amebicides, antineomatodal agents, and emetics. Ribosome 40S binder[9]. |
| Epirubicin (56390-09-1) | 2.49 ± 1.22 (32 ± 7) | 13.10 ± 5.53 (87 ± 5) | Inactive | k1.10 | Anticancer agent. DNA intercalator[7]. |
| Eprinomectin B1a (133305-88-1) | 5.33 ± 0.35 (41 ± 1) | 14.46 ± 0.98 (110 ± 6) | 13.95 ± 1.60 (108 ± 4) | k3.3 | Anthelmintic. GABA agonist[1]. |
| Ethacridine (6402-23-9) | 21.35 ± 12.03 (31 ± 11) | 18.84 ± 6.72 (75 ± 17) | Inactive | k6.20 | Antiseptic. DNA intercalator[7]. |
| Ethinylestradiol (57-63-6) | Inactive | 16.65 ± 4.89 (91 ± 20) | 51.78 ± 4.47 (36 ± 47) | k20.11 | Contraceptive. ER agonist[10]. |
| Ethylestrenol (965-90-2) | Inactive | 10.09 ± 2.97 (66 ± 11) | Inactive | k39.25 | Drug to treat testosterone deficiency. Anabolic steroid[11]. |
| Flavopiridol (146426-40-6) | Inactive | 0.248 ± 0.032 (103 ± 2) | Inactive | k1.12 | Anticancer agent. CDK inhibitor[12]. |
| Gimatecan (292618-32-7) | Inactive | 1.18 ± 0.90 (85 ± 9) | Inactive | k1.7 | Anticancer agent. Topoisomerase I inhibitor. |
| Idarubicin (57852-57-0) | Inactive | 7.53 ± 0.87 (105 ± 11) | Inactive | k1.10 | Anticancer agent. DNA intercalator[7]. |
| Ivermectin B1a (71827-03-7) | 0.57 ± 0.18 (27 ± 2) | 2.50 ± 0.52 (120 ± 8) | 14.07 ± 2.63 (124 ± 6) | k3.3 | Anthelmintic and antiparasitic for humans. GABA agonist[1]. |
| Lercanidipine (132866-11-6) | Inactive | 15.54 ± 9.59 (-71 ± 10) | Inactive | k10.12 | Cardiovascular agent. Calcium channel blocker[5]. |
| Levosimendan (141505-33-1) | Inactive | 11.02 ± 0.75 (108 ± 11) | Inactive | k14.11 | Cardiovascular agent. Calcium sensitizer[13]. |
| Mebendazole (31431-39-7) | Inactive | 1.23 ± 0.59 (66 ± 12) | Inactive | k7.10 | Anthelmintic. Tubulin binder[3]. |
| Mitomycin C (50-07-7) | Inactive | 14.52 ± 1.85 (62 ± 4) | Inactive | k7.5 | Anticancer agent. DNA damaging agent[14]. |
| Nemorubicin (108852-90-0) | Inactive | 0.21 ± 0.06 (97 ± 4) | Inactive | k1.10 | Anticancer agent. DNA intercalator[7]. |
| Nicardipine (54527-84-3) | 3.64 ± 0.84 (33 ± 7) | 16.65 ± 5.67 (116 ± 10) | 31.86 ± 15.85 (125 ± 27) | k10.12 | Cardiovascular agent. Calcium channel blocker[5]. |
| Nocodazole (31430-18-9) | Inactive | 0.32 ± 0.35 (54 ± 2) | Inactive | k7.10 | Anticancer agent. Tubulin binder[3,6]. |
| Oxibendazole (20559-55-1) | Inactive | 0.33 ± 0.25 (51 ± 3) | Inactive | k5.20 | Anthelmintic. Tubulin binder[3]. |
| Picropodophyllin (518-28-5) | Inactive | 0.075 ± 0.045 (62 ± 6) | Inactive | k3.21 | Anticancer agent. IGF-1R inhibitor[15]. |
| Selamectin (165108-07-6) | 5.98 ± 0.39 (68 ± 5) | 2.48 ± 0.82 (45 ± 6) | Inactive | k3.3 | Anthelmintic. GABA agonist[1]. |
| Surinabant | Inactive | 11.18 ± 8.93 | 34.09 ± 13.51 | k24.3 | Drug to treat nicotine addiction. $CB_1$ |

| | | | | | |
|---|---|---|---|---|---|
| (288104-79-0) | | (96 ± 16) | (66 ± 17) | | antagonist[16]. |
| Vinblastine (143-67-9) | Inactive | 0.012 ± 0.011 (59 ± 2) | Inactive | k2.26 | Anticancer agent. Tubulin binder[6]. |
| Vincristine (2068-78-2) | Inactive | 0.009 ± 0.009 (73 ± 4) | Inactive | k2.26 | Anticancer agent. Tubulin binder[6]. |
| Vinorelbine (125317-39-7) | Inactive | 0.02 ± 0.01 (73 ± 4) | 26.78 ± 4.34 (41 ± 5) | k2.26 | Anticancer agent. Tubulin binder[6]. |

ACE: Acetylcholinesterase. $CB_1$: Cannabinoid receptor type 1. CDK: Cyclin-dependent kinase. ER: Estrogen receptor. GABA: Gamma-aminobutyric acid. IGF-1R: Insulin-like growth factor 1 receptor. PDK: Pyruvate dehydrogenase kinase. PKC: Protein kinase C. RAR: Retinoic acid-activated Receptor. RXR: Retinoic X receptor. TR: Thyroid hormone receptor.

**Table S4**. PubChem BioAssay AIDs of Tox21 10K qHTS screens for AR, ERα, FXR, PPARδ, PPARγ, and VDR.

| Screen Name | BioAssay AID |
|---|---|
| AR-*bla*, agonist mode | 743053 |
| AR-*bla*, antagonist mode | 743063 |
| ERα-*bla*, agonist mode | 743077 |
| ERα-*bla*, antagonist mode | 743078 |
| FXR-*bla*, agonist mode | 743239 |
| FXR-*bla*, antagonist mode | 743240 |
| PPARδ-*bla*, agonist mode | 743227 |
| PPARδ-*bla*, antagonist mode | 743226 |
| PPARγ-*bla*, agonist mode | 743140 |
| PPARγ-*bla*, antagonist mode | 743199 |
| VDR-*bla*, agonist mode | 743241 |
| VDR-*bla*, antagonist mode | 743242 |
| Auto fluorescence at 460 nm | 720681 |
| Auto fluorescence at 535 nm | 720682 |

AID: assay identifier for PubChem's assays. AR: androgen receptor. ERα: estrogen receptor alpha. FXR: farnesoid X receptor. PPARδ: peroxisome proliferator-activated receptor delta. PPARγ: peroxisome proliferator-activated gamma. VDR: vitamin D receptor.

**Table S5**. Percentage of identified agonists and antagonists of each cluster in the 12 cell-based β-lactamase assays for FXR, AR, ERα, PPARδ, PPARγ, and VDR.

| Cluster # | % FXR agonists | % FXR antagonists | % AR agonists | % AR antagonists | % ERα agonists | % ERα antagonists | % PPARδ agonists | % PPARδ antagonists | % PPARγ agonists | % PPARγ antagonists | % VDR agonists | % VDR antagonists |
|---|---|---|---|---|---|---|---|---|---|---|---|---|
| k1.10 | 100% (6/6) | 100% (6/6) | 57% (4/7) | 57% (4/7) | 71% (5/7) | 71% (5/7) | 100% (6/6) | 100% (6/6) | 57% (4/7) | 67% (4/6) | 50% (3/6) | 50% (3/6) |
| k1.13 | 0% | 100% (2/2) | 0% | 50% (1/2) | 100% (2/2) | 50% (1/2) | 0% | 50% (1/2) | 0% | 100% (2/2) | 0% | 0% |
| k2.12 | 0% | 100% (2/2) | 0% | 50% (1/2) | 100% (2/2) | 50% (1/2) | 0% | 100% (2/2) | 100% (2/2) | 100% (2/2) | 0% | 100% (2/2) |
| k2.20 | 0% | 100% (1/1) | 0% | 100% (2/2) | 100% (2/2) | 50% (1/2) | 0% | 0% | 0% | 100% (1/1) | 0% | 100% (1/1) |
| k2.26 | 0% | 60% (3/5) | 0% | 100% (5/5) | 80% (4/5) | 100% (5/5) | 0% | 20% (1/5) | 0% | 20% (1/5) | 0% | 40% (2/5) |
| k3.3 | 78% (7/9) | 11% (1/9) | 8% (1/12) | 25% (3/12) | 0% | 8% (1/12) | 50% (2/4) | 0% | 17% (2/12) | 0% | 25% (1/4) | 0% |
| k6.1 | 50% (1/2) | 100% (2/2) | 0% | 0% | 0% | 100% (2/2) | 0% | 0% | 0% | 0% | 0% | 50% (1/2) |
| k7.9 | 13% (1/8) | 50% (4/8) | 0% | 75% (6/8) | 13% (1/8) | 13% (1/8) | 0% | 0% | 13% (1/8) | 38% (3/8) | 0% | 25% (2/8) |
| k7.10 | 20% (1/5) | 100% (5/5) | 20% (1/5) | 100% (5/5) | 20% (1/5) | 0% | 0% | 20% (1/5) | 0% | 80% (4/5) | 0% | 80% (4/5) |
| k7.15 | 33% (1/3) | 33% (1/3) | 33% (1/3) | 100% (3/3) | 100% (3/3) | 67% (2/3) | 0% | 33% (1/3) | 0% | 33% (1/3) | 0% | 0% |
| k10.12 | 43% (3/7) | 29% (2/7) | 0% | 86% (6/7) | 0% | 71% (5/7) | 0% | 0% | 0% | 14% (1/7) | 0% | 0% |
| k11.12 | 63% (7/11) | 27% (3/11) | 29% (4/14) | 29% (4/14) | 36% (5/14) | 14% (2/14) | 27% (3/11) | 9% (1/11) | 21% (3/14) | 9% (1/11) | 18% (2/11) | 0% |
| k14.11 | 0% | 67% (2/3) | 0% | 50% (2/4) | 25% (1/4) | 0% | 0% | 33% (1/3) | 0% | 67% (2/3) | 0% | 33% (1/3) |
| k18.15 | 8% (1/12) | 33% (4/12) | 0% | 7% (1/15) | 7% (1/15) | 20% (3/15) | 0% | 8% (1/12) | 0% | 33% (1/3) | 0% | 0% |
| k20.7 | 38% (3/8) | 13% (1/8) | 0% | 13% (1/8) | 0% | 0% | 0% | 0% | 0% | 0% | 0% | 0% |
| k20.11 | 0% | 50% (4/8) | 33% (3/9) | 89% (8/9) | 89% (8/9) | 11% (1/9) | 0% | 13% (1/8) | 0% | 38% (3/8) | 0% | 13% (1/8) |
| k20.18 | 0% | 40% (2/5) | 0% | 80% (4/5) | 20% (1/5) | 20% (1/5) | 0% | 0% | 0% | 100% (5/5) | 0% | 0% |
| k22.1 | 10% (1/10) | 30% (3/10) | 0% | 20% (2/10) | 0% | 30% (3/10) | 0% | 0% | 0% | 20% (2/10) | 20% (2/10) | 0% |
| k22.22 | 25% (3/12) | 17% (2/12) | 17% (2/12) | 0% | 33% (4/12) | 0% | 33% (4/12) | 0% | 33% (4/12) | 0% | 0% | 0% |
| k24.1 | 0% | 25% (4/16) | 7% (1/15) | 13% (2/15) | 33% (5/15) | 0% | 0% | 0% | 0% | 0% | 0% | 0% |
| k28.23 | 0% | 60% (3/5) | 0% | 33% (2/6) | 0% | 0% | 0% | 0% | 0% | 40% (2/5) | 0% | 0% |
| k28.24 | 0% | 75% (9/12) | 0% | 33% (4/12) | 0% | 0% | 0% | 0% | 0% | 17% (2/12) | 0% | 17% (2/12) |
| k35.18 | 0% | 26% (5/19) | 25% (5/20) | 5% (1/20) | 40% (8/20) | 5% (1/20) | 32% (6/19) | 16% (3/19) | 50% (10/20) | 21% (4/19) | 0% | 32% (6/19) |
| k37.26 | 0% | 33% (3/9) | 71% (5/7) | 43% (3/7) | 57% (4/7) | 0% | 0% | 0% | 0% | 0% | 0% | 0% |
| k38.3 | 0% | 67% (2/3) | 0% | 33% (1/3) | 0% | 33% (1/3) | 0% | 0% | 0% | 33% (1/3) | 0% | 0% |
| k38.14 | 0% | 36% (5/14) | 0% | 7% (1/14) | 14% (2/14) | 0% | 0% | 0% | 0% | 0% | 0% | 0% |
| k39.24 | 50% (3/6) | 17% (1/6) | 12% (2/17) | 18% (3/17) | 18% (3/17) | 0% | 0% | 0% | 6% (1/17) | 0% | 17% (1/6) | 0% |

The numbers of active compounds and tested compounds are reported as unique compound and in parenthesis. FXR: farnesoid X receptor. AR: androgen receptor. ERα: estrogen receptor alpha. PPARδ: peroxisome proliferator-activated receptor delta. PPARγ: peroxisome proliferator-activated gamma. VDR: vitamin D receptor.

**Table S6. qHTS protocol for FXR-*bla* and viability assays in agonist mode.**

| Step | Procedure | Parameter | Description |
|---|---|---|---|
| 1 | Plate cells | 5 μL | 5000 cells/well |
| 2 | Incubation | 5 hours | 37ºC, 5% $CO_2$ |
| 3 | Compound addition | 23 nL | CDCA and library compounds |
| 4 | Incubation time | 16 hours | 37ºC, 5% $CO_2$ |
| 5 | Substrate addition | 1 μL | $CCF_4$-AM |
| 6 | Incubation | 2 hours | Room temperature |
| 7 | FXR assay | fluorescence | Envision plate reader |
| 8 | Reagent addition | 4 μL | CellTiter Glo |
| 9 | Incubation | 30 minutes | Room temperature |
| 10 | Viability assay | luminescence | ViewLux plate reader |

$CO_2$: carbon dioxide. CDCA: chenodeoxycholic acid. FXR: farnesoid X receptor.

**Table S7. qHTS protocol for FXR-*bla* and viability assays in antagonist mode.**

| Step | Procedure | Parameter | Description |
|---|---|---|---|
| 1 | Plate cells | 5 μL | 5000 cells/well |
| 2 | Incubation | 5 hours | 37ºC, 5% $CO_2$ |
| 3 | Compound addition | 23 nL | (Z)-guggulsterone and library compounds |
| 4 | Agonist addition | 1 μL | CDCA (50 μM final concentration) |
| 5 | Incubation time | 16 hours | 37ºC, 5% $CO_2$ |
| 6 | Substrate addition | 1 μL | $CCF_4$-AM |
| 7 | Incubation | 2 hours | Room temperature |
| 8 | FXR assay | fluorescence | Envision plate reader |
| 9 | Reagent addition | 4 μL | CellTiter Glo |
| 10 | Incubation | 30 minutes | Room temperature |
| 11 | Viability assay | luminescence | ViewLux plate reader |

$CO_2$: carbon dioxide. CDCA: chenodeoxycholic acid. FXR: farnesoid X receptor.

**Figure S1**. Activities of benzoic retinoic acids (k20.7) against the FXR, AR, ERα, PPARδ, PPARγ, and VDR.

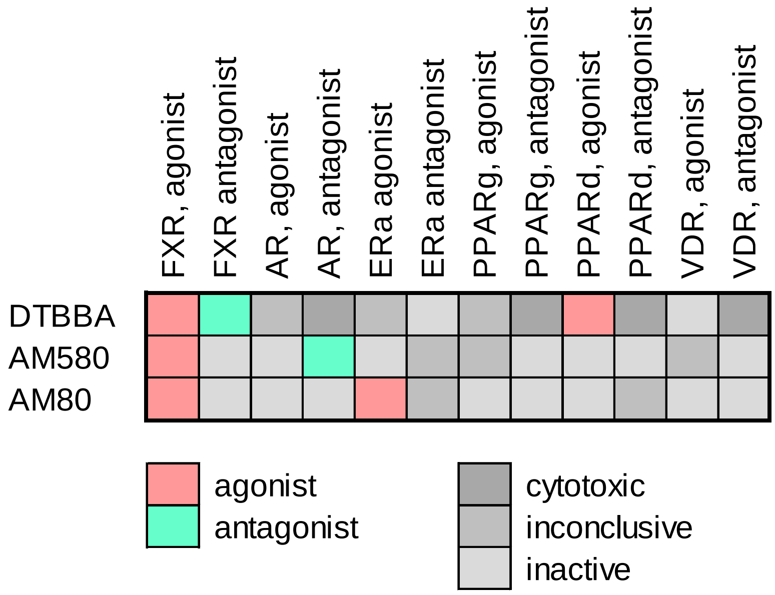


FXR: farnesoid X receptor. AR: androgen receptor. ERα: estrogen receptor alpha. PPARδ: peroxisome proliferator-activated receptor delta. PPARγ: peroxisome proliferator-activated gamma. VDR: vitamin D receptor.

**Figure S2.** Activities of dihydropyridines (k10.12 and k11.12) against FXR, AR, ERα, PPARδ, PPARγ, and VDR.

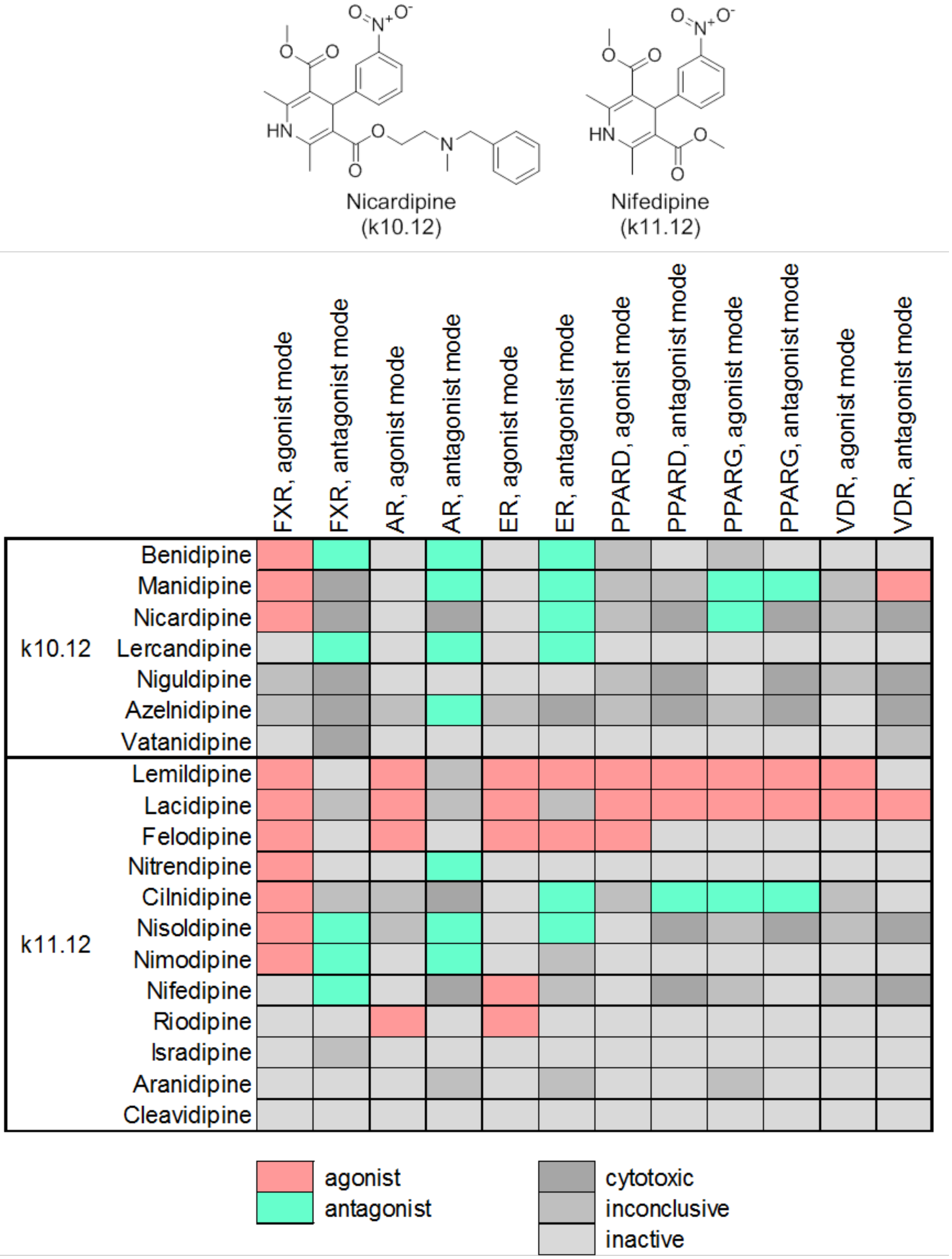


FXR: farnesoid X receptor. AR: androgen receptor. ERα: estrogen receptor alpha. PPARδ: peroxisome proliferator-activated receptor delta. PPARγ: peroxisome proliferator-activated gamma. VDR: vitamin D receptor.

**Figure S3.** Activities of anthracyclines (k1.10) against FXR, AR, ERα, PPARδ, PPARγ, and VDR.

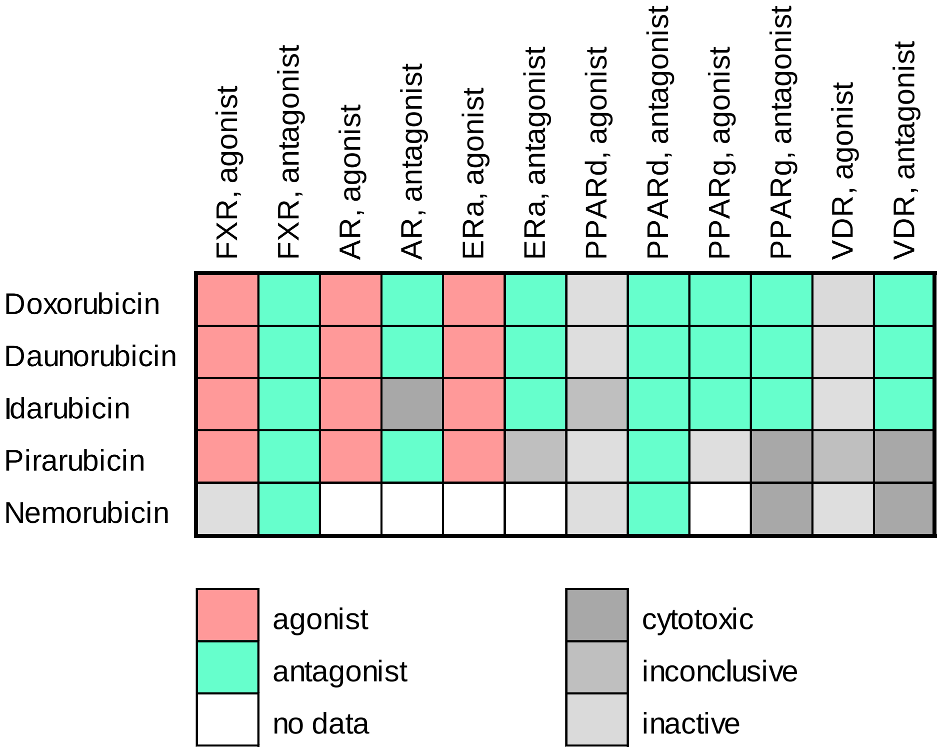


FXR: farnesoid X receptor. AR: androgen receptor. ERα: estrogen receptor alpha. PPARδ: peroxisome proliferator-activated receptor delta. PPARγ: peroxisome proliferator-activated gamma. VDR: vitamin D receptor.

**References:**


1 de Souza Spinosa, H., Gerenutti, M. & Bernardi, M. M. Anxiolytic and anticonvulsant properties of doramectin in rats: behavioral and neurochemistric evaluations. *Comp Biochem Physiol C Toxicol Pharmacol* **127**, 359-366 (2000).

2 Sobell, H. M. Actinomycin and DNA transcription. *Proc Natl Acad Sci U S A* **82**, 5328-5331 (1985).

3 Martin, R. J. Modes of action of anthelmintic drugs. *Vet J* **154**, 11-34 (1997).

4 Barrios, V. & Escobar, C. Antihypertensive and organ-protective effects of benazepril. *Expert Rev Cardiovasc Ther* **8**, 1653-1671, doi:10.1586/erc.10.159 (2010).

5 Ioan, P. *et al.* 1,4-Dihydropyridine scaffold in medicinal chemistry, the story so far and perspectives (part 1): action in ion channels and GPCRs. *Curr Med Chem* **18**, 4901-4922 (2011).

6 Lu, Y., Chen, J., Xiao, M., Li, W. & Miller, D. D. An overview of tubulin inhibitors that interact with the colchicine binding site. *Pharm Res* **29**, 2943-2971, doi:10.1007/s11095-012-0828-z (2012).

7 Rescifina, A., Zagni, C., Varrica, M. G., Pistara, V. & Corsaro, A. Recent advances in small organic molecules as DNA intercalating agents: Synthesis, activity, and modeling. *Eur J Med Chem* **74C**, 95-115, doi:10.1016/j.ejmech.2013.11.029 (2014).

8 Neri Serneri, G. G., Masotti, G., Poggesi, L., Galanti, G. & Morettini, A. Enhanced prostacyclin production by dipyridamole in man. *Eur J Clin Pharmacol* **21**, 9-15 (1981).

9 Gupta, R. S. & Siminovitch, L. The molecular basis of emetine resistance in Chinese hamster ovary cells: alteration in the 40S ribosomal subunit. *Cell* **10**, 61-66 (1977).

10 Blair, R. M. *et al.* The estrogen receptor relative binding affinities of 188 natural and xenochemicals: structural diversity of ligands. *Toxicol Sci* **54**, 138-153 (2000).

11 Saartok, T., Dahlberg, E. & Gustafsson, J. A. Relative binding affinity of anabolic-androgenic steroids: comparison of the binding to the androgen receptors in skeletal muscle and in prostate, as well as to sex hormone-binding globulin. *Endocrinology* **114**, 2100-2106, doi:10.1210/endo-114-6-2100 (1984).

12 Carlson, B. A., Dubay, M. M., Sausville, E. A., Brizuela, L. & Worland, P. J. Flavopiridol induces G1 arrest with inhibition of cyclin-dependent kinase (CDK) 2 and CDK4 in human breast carcinoma cells. *Cancer Res* **56**, 2973-2978 (1996).

13 Haikala, H. & Linden, I. B. Mechanisms of action of calcium-sensitizing drugs. *J Cardiovasc Pharmacol* **26 Suppl 1**, S10-19 (1995).

14 Lipman, R., Weaver, J. & Tomasz, M. Electrostatic complexes of mitomycin C with nucleic acids and polyanions. *Biochim Biophys Acta* **521**, 779-791 (1978).

15 E, C. *et al.* The insulin-like growth factor-I receptor inhibitor picropodophyllin-induced selective apoptosis of hepatocellular carcinoma cell through a caspase-dependent mitochondrial pathway. *Oncol Res* **21**, 103-110, doi:10.3727/096504013X13808175127324 (2014).

16 Rinaldi-Carmona, M. *et al.* SR147778 [5-(4-bromophenyl)-1-(2,4-dichlorophenyl)-4-ethyl-N-(1-piperidinyl)-1H-pyrazole-3 -carboxamide], a new potent and selective antagonist of the CB1 cannabinoid receptor: biochemical and pharmacological characterization. *J Pharmacol Exp Ther* **310**, 905-914, doi:10.1124/jpet.104.067884 (2004).
